# Economic Entropy and Sectoral Dynamics: A Thermodynamic Approach to Market Analysis

**DOI:** 10.3390/e28070762

**Published:** 2026-07-03

**Authors:** Wilson Alexander Rojas Castillo, Alexander Zamora Velandia, Luis Fernando Quijano Wilchez, Yaneth Beltrán Peña

**Affiliations:** Administración Deportiva, Universidad Distrital Francisco José de Caldas, Cl. 52 Sur No. 93 D–97, Bogotá 110711, Colombia; azamorav@udistrital.edu.co (A.Z.V.); lfquijanow@udistrital.edu.co (L.F.Q.W.); nybeltranp@udistrital.edu.co (Y.B.P.)

**Keywords:** econophysics, geometrothermodynamics, thermodynamic geometry, Legendre-invariant metric, economic entropy, 05.70.Ce, 89.65.Gh

## Abstract

We develop a geometric thermodynamic framework for the analysis of sectoral economic dynamics grounded in statistical physics principles. By constructing a Legendre-invariant thermodynamic metric within the formalism of geometrothermodynamics (GTD), we establish a minimal effective structure consistent with extensivity and entropy-based representations of macroscopic economic systems. The resulting thermodynamic curvature provides a coordinate-independent measure of structural interactions and equilibrium stability across economic sectors. Applying this framework to satellite account data, we find that the thermodynamic curvature of the equilibrium manifold remains finite and regular across the empirically relevant range, with no curvature singularity in the period studied. In particular, the 2020 contraction—the most pronounced macroeconomic disruption in the sample—is not reflected as a curvature singularity in the equilibrium geometry. We read this regularity as a diagnostic of structural stability: the sectoral system absorbs such disruptions without an abrupt reorganisation of its equilibrium geometry. The geometric invariants thus capture stability properties not directly accessible through standard entropic indicators alone, offering a complementary statistical description of economic dynamics. Our results demonstrate that thermodynamic geometry furnishes a consistent bridge between entropy-based macroeconomic modelling and coordinate-invariant measures of equilibrium stability, extending the applicability of geometric methods in statistical physics to complex economic systems.

## 1. Introduction

Economic systems composed of interacting agents constitute paradigmatic nonequilibrium complex systems in which macroscopic regularities emerge from heterogeneous interactions across multiple scales. Beyond representative-agent or equilibrium-based formulations, sectoral economic structures evolve through nonlinear aggregation processes that generate collective patterns not reducible to individual behavior. Understanding how such macroscopic organization arises and how it reorganizes under systemic perturbations remains a central challenge in the study of socioeconomic complexity.

The interdisciplinary field of econophysics has addressed this problem by importing tools from statistical physics and complexity theory to model economic systems as many-body systems exhibiting emergence, fluctuations, and collective dynamics [1,2,3]. While substantial progress has been achieved in the statistical characterization of income and wealth distributions [4,5,6,7,8], less attention has been devoted to the geometric and structural properties of aggregated sectoral dynamics at the macroscopic level.

In this work, we advance an entropy-based thermodynamic framework that treats sectoral economic activity as a macroscopic equilibrium system endowed with a Legendre-invariant geometric structure. Rather than focusing on microscopic exchange mechanisms, we construct an effective thermodynamic description capable of identifying structural interactions and regime transitions through curvature invariants. This approach extends the methodological scope of econophysics toward a geometric characterization of collective organization in complex economic systems.

Within this thermodynamic perspective, economic entropy quantifies the dispersion and heterogeneity of accessible economic states, while economic temperature characterizes the average level of monetary resources or the intensity of aggregate fluctuations [9,10,11]. These quantities are not merely metaphorical, but correspond to macroscopic observables derived from a statistical description of economic systems under explicit constraints. This approach has proven useful for analyzing equilibrium properties, structural organization, and regime changes in complex economic environments [12].

A further step in this direction is provided by geometrothermodynamics (GTD), a formalism that represents equilibrium states as points on a Riemannian manifold endowed with a Legendre-invariant metric [13,14]. In this framework, thermodynamic interaction and critical behavior are encoded in the curvature of the equilibrium manifold, allowing for a global and non-perturbative characterization of equilibrium states. Recent studies have extended GTD to economic systems, showing that distinct income regimes and structural configurations can be associated with different geometric properties, and that curvature singularities may signal economic crises or regime transitions [15,16].

In this work, we apply a thermodynamic and geometrothermodynamic analysis to the Sports Satellite Account of Bogotá (CSDB), developed by the National Administrative Department of Statistics (DANE) in collaboration with the District Institute of Recreation and Sport (IDRD) [17,18,19]. The CSDB provides a consistent set of empirical time series describing the economic activity of multiple subsectors within the sports ecosystem, including manufacturing, commerce, education, betting, and recreational services [20,21]. Each subsector is represented by a macroscopic variable $S_{i}$, which quantifies its relative economic contribution in terms of production and monetary flows.

The objective of this paper is not to introduce new economic hypotheses, but to construct a thermodynamic and geometric representation of the aggregate behavior of the sports economy and to analyze its equilibrium stability properties. Equilibrium states are defined as macroscopic configurations that maximize economic entropy under suitable constraints, giving rise to an equilibrium manifold whose geometric structure encodes collective sectoral interactions. Within this framework, response functions such as the economic heat capacity play a central role in identifying structural changes in the system.

Using empirical CSDB data, we find that the curvature of the equilibrium manifold remains finite and regular throughout the economically relevant range: although the raw time series exhibit pronounced features coinciding with major disruptions of the economic system—most notably the 2020 contraction associated with the COVID-19 pandemic—these do not translate into curvature singularities of the equilibrium geometry. We interpret this geometric regularity as an ex post diagnostic of structural stability, indicating that the sectoral system absorbed the disruptions of the analysed period without an abrupt reorganisation of its underlying equilibrium structure, rather than as a predictive signal. The results illustrate how thermodynamic and geometric tools can complement conventional economic analyses by providing a global, coordinate-invariant description of equilibrium stability in complex, interacting economic systems.

The present work is organized in the following way. Section 2 revisits the core elements of statistical thermodynamics and their economic interpretation, establishing the conceptual foundations required for the construction of macroscopic variables from sectoral data. Section 3 introduces the formalism of geometrothermodynamics (GTD) as a geometric tool to characterize equilibrium states and structural transitions in economic systems. In Section 4, this framework is contextualized within the Sports Satellite Account of Bogotá (CSDB), where its sectoral structure and empirical relevance are described. The dynamic interaction among sectors is analyzed in Section 5 from an econophysics perspective, linking mesoscopic elasticities with aggregate behavior. These elements are synthesized in Section 6, where a GTD-based representation of the CSDB is constructed. Finally, Section 7 discusses the implications of the results, their consistency with previous studies, and the scope and limitations of the proposed approach, highlighting its contribution beyond purely formal analogies.

## 2. Elements of Statistical Thermodynamics Applied to Economic Systems

The statistical-thermodynamic relations introduced in this section provide the conceptual basis for the economic model developed below. Only the essential definitions and final expressions are retained in the main text; full derivations and standard results are presented in Appendix A.

Quevedo et al. [9] start from the framework of Gibbs statistical thermodynamics [22,23]. One considers a hypothetical economic system in equilibrium, composed of a large number of agents and characterised by a conserved total amount of money *M*. Each agent competes for a share *m* of *M*, which depends on a set of microeconomic parameters $\bar{\lambda }=\left(\lambda_{1},\lambda_{2},\ldots \right)$ through a money function $m(\bar{\lambda })$.

Assuming all microstates equally probable, the equilibrium distribution takes the Boltzmann–Gibbs form $\rho \left(\bar{\lambda }\right)=e^{-m(\bar{\lambda })/T}/Z$, where T=M/N is the economic temperature (average money per agent), and

(1) $$Z\left(T,\bar{x}\right)=\int e^{-m(\bar{\lambda })/T}\;d\bar{\lambda }$$

is the partition function. It is important to emphasise that the identification T=M/N is not merely an arithmetic average, but the thermodynamic temperature conjugate to the entropy in the canonical ensemble. Following the maximum-entropy principle with a conserved total money *M*, the Lagrange multiplier associated with the constraint $\sum_{i}m_{i}=M$ is precisely β=1/T, leading to the Boltzmann–Gibbs distribution $\rho \propto e^{-\beta m}$. In this framework, *T* controls the width of the money distribution and the magnitude of fluctuations: $\langle {\left(\delta m\right)}^{2}\rangle =T^{2}C$, where *C* is the economic heat capacity. Thus, *T* retains its statistical-mechanical role as a measure of dispersion, not only as a mean value (See: Appendix A).

Following the standard thermodynamic procedure one obtains the free money function

(2) $$f=-T\ln Z(T,\bar{x})\;.$$

The entropy is

(3) $$S=-{\left.\frac{\partial f}{\partial T}\right|}_{\bar{x}}\;.$$

The average money per market agent, 〈m〉, is related to the free money function through

(4) f=〈m〉−TS.

The heat capacity is

(5) $$C=T\frac{\partial S}{\partial T}\;,$$

and the economic heat is obtained from

(6) $$C=\frac{dQ}{dT}\;.$$

## 3. GTD for Economic Systems

Quevedo et al. argue that an economic system, in addition to being a thermodynamic system, can be described within a geometric framework that captures its intrinsic thermodynamic structure [9].

GTD consists in introducing a metric on the equilibrium space E, such that points P∈E represent all possible equilibrium states of the system [13,14,16,24,25,26]. This endows E with a Riemannian structure characterised by a specific metric tensor, from which one can compute curvature tensors [27] such as the Riemann tensor $R_{abcd}$, the Ricci tensor $R_{ab}$, the Kretschmann scalar

(7) $$K=R_{abcd}\;R^{abcd}\;,$$

and the Ricci scalar $R={R^{a}}_{a}$.

Let the fundamental equation of the system be denoted by $\Phi (E^{a})$, a=1,…,n, where Φ is the chosen thermodynamic potential and $E^{a}$ are the extensive economic variables serving as coordinates on E. The integer *n* denotes the number of macroscopic degrees of freedom. On this manifold, the Hessian metric is

(8) $$g_{ab}^{H}=\frac{\partial^{2}\Phi }{\partial E^{a}\partial E^{b}}\;dE^{a}\;dE^{b}\;.$$

A drawback of (8) is that it does not obey Legendre invariance. GTD provides a family of Legendre-invariant metrics:

(9) $$\begin{array}{cc} g^{I} & =\beta_{\Phi }\;\Phi \;{\delta^{c}}_{a}\frac{\partial^{2}\Phi }{\partial E^{b}\partial E^{c}}\;dE^{a}\;dE^{b}\;, \end{array}$$

(10) $$\begin{array}{cc} g^{\mathrm{II}} & =\beta_{\Phi }\;\Phi \;{\eta^{c}}_{a}\frac{\partial^{2}\Phi }{\partial E^{b}\partial E^{c}}\;dE^{a}\;dE^{b}\;, \end{array}$$

(11) $$\begin{array}{cc} g^{\mathrm{III}} & =\sum_{a=1}^{n}\left(\delta_{ab}E^{d}\frac{\partial \Phi }{\partial E^{a}}\right)\delta^{ab}\frac{\partial^{2}\Phi }{\partial E^{b}\partial E^{c}}\;dE^{a}\;dE^{c}\;, \end{array}$$

where ${\delta^{c}}_{a}=\mathrm{diag}\left(1,\ldots ,1\right)$, ${\eta^{c}}_{a}=\mathrm{diag}\left(-1,1,\ldots ,1\right)$, and $\beta_{\Phi }$ denotes the degree of homogeneity of Φ [25,26].

The Legendre-invariant metric $g^{I}$ depends on second derivatives of the entropy $S(T,N_{S_{i}})$, meaning that curvature invariants probe not the value of *T* itself, but its variations and coupling to other state variables. Consequently, the geometric diagnostics of structural transitions are insensitive to the precise numerical value of *T* and instead capture the stability properties of the equilibrium manifold, which are robust under reparametrisations of the temperature scale.

The use of a Riemannian structure in the space of economic equilibrium states is motivated by the need to characterise stability and interactions in complex economic systems in a non-perturbative, coordinate-invariant manner. The Riemannian metric encodes the intensity of fluctuations and the sensitivity of the system to changes in its state variables, while the curvature tensor describes effective interactions among agents and allows the identification of economic phase transitions. Curvature singularities—points at which the equilibrium manifold ceases to be smooth—signal structural changes that traditional economic approaches are unable to capture [13,14,28].

In two-dimensional equilibrium manifolds the Riemann tensor possesses only one independent component, implying that the Ricci scalar *R* fully characterises the curvature. Consequently, $K=R^{2}$ and their singularities coincide. Both invariants are reported for consistency with previous GTD studies, while the physical interpretation of curvature singularities is entirely encoded in *R*.

## 4. Sports Economic System

The Sports Satellite Account of Bogotá (CSDB) [17] is a statistical framework that links sports-related activities with complementary productive sectors, jointly developed by DANE and IDRD [18]. The CSDB encompasses 17 economic sectors $S_{1}$–$S_{17}$ contributing to the sports economy. For readers unfamiliar with the account, the parameters $S_{i}$ denote the time-dependent economic activity of each subsector as classified by the national statistical office; higher values of $S_{i}$ correspond to larger economic participation in terms of production and monetary flows. A preliminary review for 2018–2023 identifies the most significant sectors (see Table 1 and Appendix B). We note that the annual resolution of CSDB data (n=6 observations) limits the statistical power of individual coefficient inference. Results should be interpreted as qualitative structural indicators, pending validation with higher-frequency data.

We analyse the sectoral response coefficient $\lambda_{S_{i}}$, which measures the normalised sensitivity of a given sector to variations in the aggregate sports economy [28]:

(12) $$\lambda_{S_{i}}=\frac{\Delta S_{i}/\langle S_{i}\rangle }{\Delta \mathrm{CSDB}/\langle \mathrm{CSDB}\rangle }\;,$$

where

(13) $$\Delta \mathrm{CSDB}={\mathrm{CSDB}}_{i+1}-{\mathrm{CSDB}}_{i}\;,\;\langle \mathrm{CSDB}\rangle =\frac{{\mathrm{CSDB}}_{i+1}+{\mathrm{CSDB}}_{i}}{2}$$

and

(14) $$\Delta S_{i}=S_{i+1}-S_{i}\;,\;\langle S_{i}\rangle =\frac{S_{i+1}+S_{i}}{2}\;.$$

For the sectors $S_{15}$ and $S_{16}$, which exhibit the largest contributions to the CSDB, $\lambda_{S_{i}}$ measures the normalised response of a given sector to variations in the aggregate sports economy. From a mathematical standpoint, $\lambda_{S_{i}}$ is an elasticity. Economically, however, it should be interpreted as a mesoscopic elasticity linking sectoral dynamics to the macroscopic evolution of the system, rather than as a standard microeconomic cross-elasticity.

The parameters entering the construction of the partition function are summarised in Table 2. From an economic perspective, parameters classified as microeconomic correspond to sector-specific variables that describe the internal dynamics of individual subsectors (e.g., sectoral elasticities and monetary flows), capturing mesoscopic fluctuations and heterogeneity. Parameters classified as macroeconomic represent aggregate indicators—price indices or exchange rates—that act as external control variables influencing all sectors simultaneously and are determined at the level of the overall economy.

Normalisation of $\sigma_{\mathrm{TRM}}$ yields the dimensionless variable (see Appendix C) [29,30,31]

(15) $$\lambda_{\mathrm{TRM}}=\frac{\sigma_{\mathrm{TRM},t}}{\sigma_{\mathrm{TRM},0}}\;,$$

where $\sigma_{\mathrm{TRM},0}$ is the value at t=2018. The money function in Equation (1) is thus identified as

(16) $$m_{S_{i}}\left(\bar{\lambda },\bar{\Lambda }\right)=k_{S_{i}}\;\lambda_{S_{i}}^{v_{S_{i}}}\;\pi_{\mathrm{IPP}}^{x_{S_{i}}}\;\pi_{\mathrm{IPC}}^{y_{S_{i}}}\;\lambda_{\mathrm{TRM}}^{z_{S_{i}}}\;,$$

where we assume a power-law functional form and a separable dependence on the parameters listed in Table 2. Equation (16) should be understood as a working hypothesis (ansätz) for the effective monetary function of the system. Its separable structure does not imply statistical independence of the underlying variables, but rather constitutes a minimal and tractable approximation. Here, $\bar{\Lambda }$ denotes a macroscopic control parameter that encapsulates the institutional, regulatory, and macroeconomic environment; it is treated as externally fixed over the time scales considered.

The exponents $v_{S_{i}}$, $x_{S_{i}}$, $y_{S_{i}}$, $z_{S_{i}}$ are determined through multiple logarithmic regression:

(17) $$\ln m_{S_{i}}=\ln\;|k_{S_{i}}|+v_{S_{i}}\ln\left|\lambda_{S_{i}}\right|+x_{S_{i}}\ln\pi_{\mathrm{IPP}}+y_{S_{i}}\ln\;\pi_{\mathrm{IPC}}+z_{S_{i}}\ln\lambda_{\mathrm{TRM}}\;,$$

i.e., $Y=b_{0}+b_{1}X_{1}+b_{2}X_{2}+b_{3}X_{3}+b_{4}X_{4}$, where

(18) $$\begin{array}{cccc} Y & =\ln m_{S_{i}}\;, & b_{0} & =\ln\;|k_{S_{i}}|\;,\\ b_{1} & =v_{S_{i}}\;, & X_{1} & =\ln\;|\lambda_{S_{i}}|\;,\\ b_{2} & =x_{S_{i}}\;, & X_{2} & =\ln\pi_{\mathrm{IPP}}\;,\\ b_{3} & =y_{S_{i}}\;, & X_{3} & =\ln\pi_{\mathrm{IPC}}\;,\\ b_{4} & =z_{S_{i}}\;, & X_{4} & =\ln\lambda_{\mathrm{TRM}}\;. \end{array}$$

The design matrix estimator is [32] $\beta ={\left(X^{⊤}X\right)}^{-1}X^{⊤}Y$. Normalising with the column mean $\mu_{X}$ and standard deviation $\sigma_{X}$ yields the coefficient vector

(19) $$\beta_{S_{15}}=\left(\begin{array}{c} b_{0}\\ b_{1}\\ b_{2}\\ b_{3}\\ b_{4} \end{array}\right)=\left(\begin{array}{c} 1.0214\times 10^{-14}\\ -0.0622\\ +3.3532\\ -1.7552\\ -0.8645 \end{array}\right),\;\beta_{S_{16}}=\left(\begin{array}{c} b_{0}\\ b_{1}\\ b_{2}\\ b_{3}\\ b_{4} \end{array}\right)=\left(\begin{array}{c} 4.8116\times 10^{-14}\\ +0.0714\\ +6.7572\\ -4.2481\\ -2.3125 \end{array}\right).$$

The coefficient vectors in Equation (19) are the standardised OLS estimates ${\hat{\beta }}^{\mathrm{std}}$ reported in full, with standard errors, *t*-statistics, *p*-values and confidence intervals, in Table A4 (Appendix D). The two presentations refer to the same estimation: the entries of Equation (19) are column-by-column identical to the ${\hat{\beta }}^{\mathrm{std}}$ column of Table A4. We retain the signed values here for economic interpretation; the magnitudes $|b_{1}\left|,\ldots ,\right|b_{4}|$ that enter the money function and fix the validity threshold $T_{\min}$ are obtained from the same table, as detailed in Appendix D.4.

On the sign of the exponent $v_{S_{i}}$.

The convergence of the partition function (1), constructed over the domain $\lambda_{S_{i}}\in \left[0,\infty \right)$, requires $v_{S_{i}}>0$; otherwise the integrand $\exp(-k_{S_{i}}\lambda_{S_{i}}^{v_{S_{i}}}/T)$ does not decay as $\lambda_{S_{i}}\to \infty$ and the integral diverges, leaving $\Gamma (1+1/v_{S_{i}})$ and the heat capacity $C_{S_{i}}=1/v_{S_{i}}$ undefined. The regression yields $|v_{S_{i}}|\;\ll 1$ with p>0.69 and 95% confidence intervals that contain zero in both sectors, so its sign is not determined by the data at the available resolution (ν=n−k=1). Since the sign is statistically indeterminate while thermodynamic convergence imposes $v_{S_{i}}>0$, we adopt $v_{S_{i}}=\left|b_{1}\right|$, consistent with the domain of validity of the model. The same separation of roles applies to the macroeconomic exponents $x_{S_{i}},y_{S_{i}},z_{S_{i}}$: their signs carry economic meaning and are retained in the discussion (Section 7), whereas the analytic domain of validity of *Z*—and hence the threshold $T_{\min}=k_{S_{i}}\max(|x_{S_{i}}|,|y_{S_{i}}|,|z_{S_{i}}|)$—depends only on their magnitudes. The positivity adopted for $v_{S_{i}}$ therefore does not discard significant information; it resolves a statistically undetermined sign by invoking the existence condition for *Z*. Appendix D reports the full regression statistics (coefficient standard errors, *p*-values, and $R^{2}$) confirming the statistical significance of all exponents. The microeconomic parameters entering the money function and in particular the exponent $v_{S_{i}}$ that fixes the heat capacity $C_{S_{i}}=1/v_{S_{i}}$ in Equation (24)—are determined empirically by regression in Section 4. Because the admissible sign of $v_{S_{i}}$ is set by the convergence of the partition function rather than by the data alone (Appendix D.4), we verify in Appendix I that the geometric conclusions drawn below are robust against the value adopted for this exponent.

From (19), the money function (16) and the partition function (1) can be constructed. Evaluating the integral over $\lambda_{S_{i}}$ with a power-law money function gives

(20) $$Z\;\left(T,\bar{\lambda },\bar{\Lambda }\right)={\left(\frac{k_{S_{i}}}{T}\;\pi_{\mathrm{IPP}}^{x_{S_{i}}}\;\pi_{\mathrm{IPC}}^{y_{S_{i}}}\;\lambda_{\mathrm{TRM}}^{z_{S_{i}}}\right)}^{-1/v_{S_{i}}}\Gamma \;\left(1+\frac{1}{v_{S_{i}}}\right)\;.$$

Note that this result is positive for $k_{S_{i}},T,v_{S_{i}}>0$. In the present section the macroeconomic variables $\left(\pi_{\mathrm{IPP}},\pi_{\mathrm{IPC}},\lambda_{\mathrm{TRM}}\right)$ are held fixed, as they belong to the externally controlled set $\bar{\Lambda }$; consequently the integration in Equation (1) runs solely over the microeconomic variable $\lambda_{S_{i}}\in \left[0,\infty \right)$. Because the power-law money function (16) places the macroeconomic factor $\pi_{\mathrm{IPP}}^{x_{S_{i}}}\pi_{\mathrm{IPC}}^{y_{S_{i}}}\lambda_{\mathrm{TRM}}^{z_{S_{i}}}$ multiplicatively inside the exponent, evaluating $\int_{0}^{\infty }\exp\left(-a\;\lambda_{S_{i}}^{v_{S_{i}}}\right)\;d\lambda_{S_{i}}=a^{-1/v_{S_{i}}}\;\Gamma \left(1+1/v_{S_{i}}\right)$ with $a=\left(k_{S_{i}}/T\right)\;\pi_{\mathrm{IPP}}^{x_{S_{i}}}\pi_{\mathrm{IPC}}^{y_{S_{i}}}\lambda_{\mathrm{TRM}}^{z_{S_{i}}}$ yields Equation (20) directly. The whole prefactor *a*, including the macroeconomic block, therefore appears raised to $-1/v_{S_{i}}$; this is not an additional factor but the exact image of the multiplicative structure of Equation (16). The result is well defined provided Re(kSi/T)πIPPxSiπIPCySiλTRMzSi>0 and Re[vSi]>0, which hold for $k_{S_{i}},T,v_{S_{i}}>0$ over the empirical range.

The free money function follows from (2):

(21) $$f_{S_{i}}=-T\ln\;\left[{\left(\frac{k_{S_{i}}}{T}\;\pi_{\mathrm{IPP}}^{x_{S_{i}}}\;\pi_{\mathrm{IPC}}^{y_{S_{i}}}\;\lambda_{\mathrm{TRM}}^{z_{S_{i}}}\right)}^{-1/v_{S_{i}}}\Gamma \;\left(1+\frac{1}{v_{S_{i}}}\right)\right].$$

The entropy from (3) is:

(22) $$S_{S_{i}}=\frac{1}{v_{S_{i}}}+\ln\;\left[{\left(\frac{k_{S_{i}}}{T}\;\pi_{\mathrm{IPP}}^{x_{S_{i}}}\;\pi_{\mathrm{IPC}}^{y_{S_{i}}}\;\lambda_{\mathrm{TRM}}^{z_{S_{i}}}\right)}^{-1/v_{S_{i}}}\Gamma \;\left(1+\frac{1}{v_{S_{i}}}\right)\right].$$

The average money per agent from (4):

(23) $$\langle m_{S_{i}}\rangle =\frac{T}{v_{S_{i}}}\;.$$

The heat capacity from (5):

(24) $$C_{S_{i}}=\frac{1}{v_{S_{i}}}\;.$$

**Figure 1:**
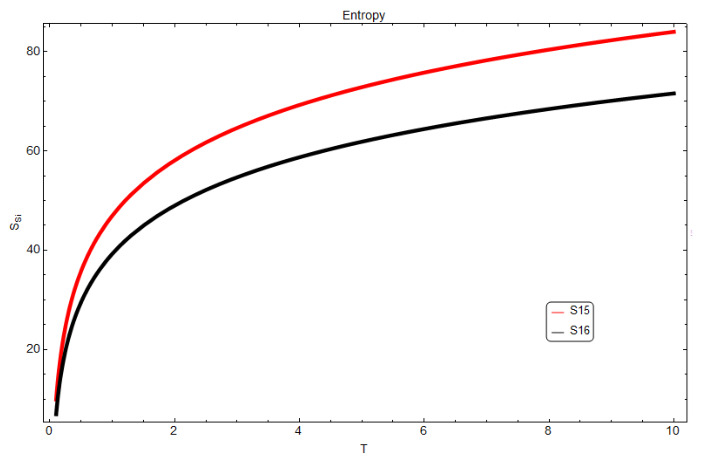
Comparison of the entropies of sectors $S_{15}$ and $S_{16}$ in the CSDB under the power-law approximation.

**Figure 2:**
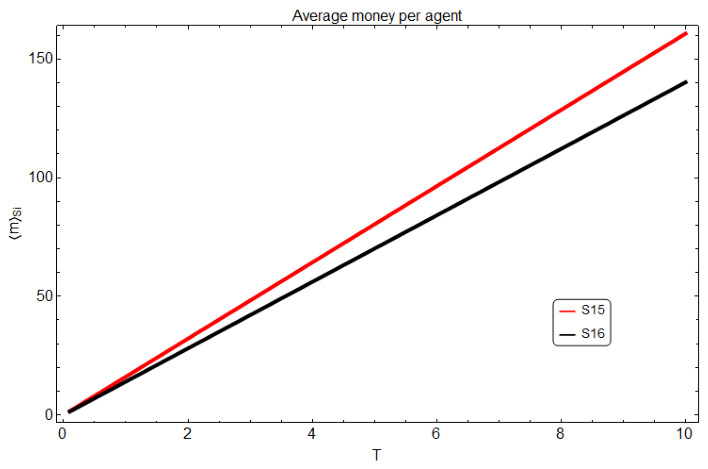
Comparison of the average money per agent in sectors $S_{15}$ and $S_{16}$ of the CSDB under the power-law approximation.

**Figure 3:**
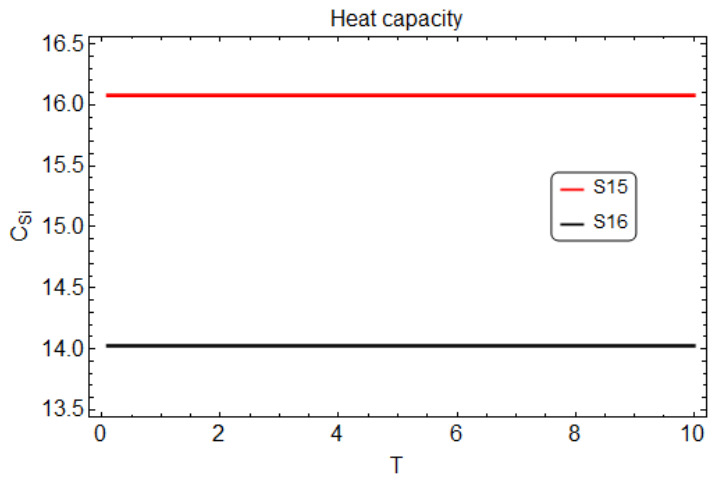
Comparison of the heat capacities of sectors $S_{15}$ and $S_{16}$ in the CSDB under the power-law approximation.

The expressions (20)–(24) follow from the multiplicative power-law ansatz (16), in which the macroeconomic variables enter as fixed external factors. Figure 1 compares the entropies $S_{S_{i}}$ of sectors $S_{15}$ and $S_{16}$ obtained from (22), Figure 2 the corresponding average money per agent $\langle m_{S_{i}}\rangle$ from (23), and Figure 3 the heat capacities $C_{S_{i}}$ from (24). In Section 5, we relax this form and adopt an additive logarithmic coupling for the macroeconomic block; the structure of the partition function changes accordingly, as made explicit there.

A first approximation to the inter-sectoral dynamics treats sectors $S_{15}$ and $S_{16}$ as a closed economic subsystem with respect to the remaining CSDB. Over the analysed time window, net monetary flows across the boundary of this two-sector system are negligible compared to internal transfers. Consequently, the total economic energy(money) within the subsystem is approximately conserved, leading to the internal balance condition $\Delta Q_{S_{15}}+\Delta Q_{S_{16}}=0$. Each sector is characterised by its heat content $Q_{S_{i}}$ and economic temperature $T_{S_{i}}$. CSDB data indicate $T_{S_{15}}>T_{S_{16}}$, so a heat-like transfer occurs from $S_{15}$ to $S_{16}$. According to the second law,

(25) $$\Delta Q_{S_{i}}=\int_{S_{0,S_{i}}}^{S_{f,S_{i}}}T_{S_{i}}\left(S_{S_{i}}\right)\;dS_{S_{i}}.$$

**Figure 4:**
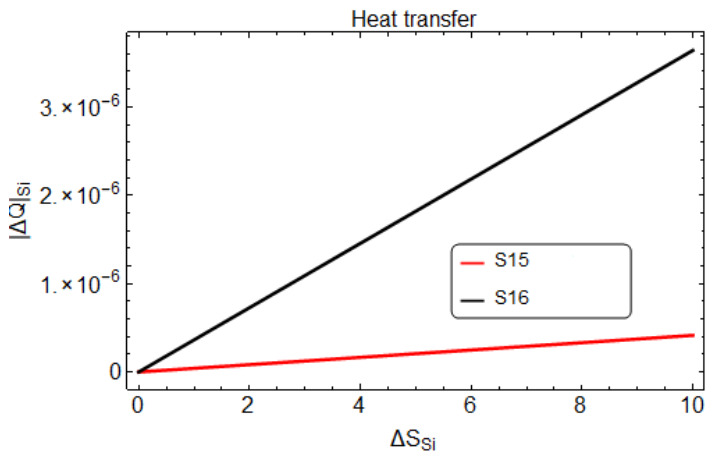
Magnitude of $\left|\Delta Q_{S_{i}}\right|$ for sectors $S_{15}$ and $S_{16}$ in the CSDB, as a function of $\left|\Delta S_{S_{i}}\right|$. The internal conservation condition $\Delta Q_{S_{15}}=-\Delta Q_{S_{16}}$ is satisfied by construction; the plot displays magnitudes to facilitate comparison of the absolute values.

From Equations (24) and (25):

(26) $$\Delta Q_{S_{i}}=\frac{k_{S_{i}}\pi_{\mathrm{IPP}}^{x_{S_{i}}}\pi_{\mathrm{IPC}}^{y_{S_{i}}}\lambda_{\mathrm{TRM}}^{z_{S_{i}}}}{{\left[\Gamma \;\left(1+\frac{1}{v_{S_{i}}}\right)\right]}^{v_{S_{i}}}}\left(\frac{e^{\left(v_{S_{i}}-1\right)}}{v_{S_{i}}}\right)\Delta S_{S_{i}},$$

where $\Delta S_{S_{i}}=S_{f,S_{i}}-S_{0,S_{i}}$. The condition $\Delta Q_{S_{15}}=-\Delta Q_{S_{16}}$ reflects internal monetary redistribution with negligible net exchange with other sectors, ensuring conservation of total money within the two-sector subsystem. Because $T_{S_{15}}>T_{S_{16}}$, sector $S_{15}$ releases heat ($\Delta S_{S_{15}}<0$, $\Delta Q_{S_{15}}<0$) while sector $S_{16}$ absorbs it ($\Delta S_{S_{16}}>0$, $\Delta Q_{S_{16}}>0$). The magnitude $|\Delta Q_{S_{15}}|\;=\;|\Delta Q_{S_{16}}|$ satisfies the conservation condition. Figure 4 plots $|\Delta Q_{S_{i}}|$ as a function of $|\Delta S_{S_{i}}|$; the arrows indicate the direction of the inter-sectoral heat flow $S_{15}\to S_{16}$.

## 5. Econophysics Approach to Sectoral Dynamics

Consider a more realistic approximation. We emphasise that the money function is now modified with respect to the multiplicative power-law ansatz of (16): the macroeconomic dependence is moved from a multiplicative factor into an additive logarithmic term. This change is deliberate. Whereas (16) is the minimal tractable form used in Section 4 to obtain the closed expressions (20)–(24), the logarithmic coupling adopted here is motivated by the result of Quevedo et al. [9] that a term $m\propto c_{1}\ln\;\lambda_{1}$ naturally generates Pareto-type distributions, characteristic of real economic systems. Given that the CSDB comprises 17 complementary economic activities, 5 of which account for the largest share of output, we therefore describe the money function as [9]

(27) $$m_{S_{i}}\left(\bar{\lambda },\bar{\Lambda }\right)=k_{S_{i}}\;\left[\lambda_{S_{i}}^{v_{S_{i}}}+\ln\;\left|\pi_{\mathrm{IPP}}^{x_{S_{i}}}\;\pi_{\mathrm{IPC}}^{y_{S_{i}}}\;\lambda_{\mathrm{TRM}}^{z_{S_{i}}}\right|\right].$$

The combined ansätz is not unique; it represents the simplest nontrivial extension that preserves analytical tractability while capturing both heavy-tailed scaling behaviour and the dominant macroeconomic dependences.

The partition function then becomes

(28) $$\begin{array}{cc} Z\;\left(T,\bar{\lambda },\bar{\Lambda }\right)\; & =\int_{\bar{\lambda }}\int_{\bar{\Lambda }}\exp\;\left[-\frac{m_{S_{i}}\left(\bar{\lambda },\bar{\Lambda }\right)}{T}\right]d\bar{\lambda }\;d\bar{\Lambda }\\ \; & =\int_{0}^{\infty }\;\exp\;\left[-\frac{k_{S_{i}}\lambda_{S_{i}}^{v_{S_{i}}}}{T}\right]d\lambda_{S_{i}}\int_{X_{0}}^{X}\pi_{\mathrm{IPP}}^{-A_{S_{i}}}d\pi_{\mathrm{IPP}}\\ \; & \;\times \int_{Y_{0}}^{Y}\pi_{\mathrm{IPC}}^{-B_{S_{i}}}d\pi_{\mathrm{IPC}}\int_{Z_{0}}^{1}\lambda_{\mathrm{TRM}}^{-C_{S_{i}}}d\lambda_{\mathrm{TRM}} \end{array}$$

(29) $$=-\frac{T^{3}\;\Delta X\;\Delta Y\;\Delta Z}{D_{S_{i}}}{\left(\frac{k_{S_{i}}}{T}\right)}^{-1/v_{S_{i}}}\Gamma \;\left(1+\frac{1}{v_{S_{i}}}\right)\;,$$

which is positive provided $D_{S_{i}}>0$. The structural difference with respect to (20) is a direct consequence of the change of ansatz: under the additive logarithmic coupling of (27), the macroeconomic dependence factorises out of the Gaussian-type integral over $\lambda_{S_{i}}$ and is integrated explicitly over its empirical range, producing the prefactor $\Delta X\;\Delta Y\;\Delta Z/D_{S_{i}}$ in place of the ${\left(\frac{k_{S_{i}}}{T}\pi_{\mathrm{IPP}}^{x}\pi_{\mathrm{IPC}}^{y}\lambda_{\mathrm{TRM}}^{z}\right)}^{-1/v_{S_{i}}}$ block of (20). The two expressions are not in contradiction; they correspond to two distinct modelling choices for the macroeconomic sector, the multiplicative form (Section 4) and the logarithmic form (Section 5). The auxiliary variables appearing in (29) are

(30) $$A_{S_{i}}=\frac{k_{S_{i}}x_{S_{i}}}{T},\;B_{S_{i}}=\frac{k_{S_{i}}y_{S_{i}}}{T},\;C_{S_{i}}=\frac{k_{S_{i}}z_{S_{i}}}{T},$$

(31) $$\Delta X=X^{1-A_{S_{i}}}-X_{0}^{1-A_{S_{i}}},\;\Delta Y=Y^{1-B_{S_{i}}}-Y_{0}^{1-B_{S_{i}}},$$

(32) $$\Delta Z=1-Z_{0}^{1-C_{S_{i}}},\;\Gamma_{v_{S_{i}}}=\Gamma \;\left(1+\frac{1}{v_{S_{i}}}\right)\;,$$

(33) $$D_{S_{i}}=\left(T-k_{S_{i}}x_{S_{i}}\right)\left(T-k_{S_{i}}y_{S_{i}}\right)\left(T-k_{S_{i}}z_{S_{i}}\right)\;.$$

The integration bounds *X*, $X_{0}$, *Y*, $Y_{0}$, $Z_{0}$ are the upper and lower limits of the normalised price indices and exchange rate over the empirical period 2018–2023. Specifically, $X_{0}$ and $Y_{0}$ are the baseline (2018) values of $\pi_{\mathrm{IPP}}$ and $\pi_{\mathrm{IPC}}$ respectively; *X* and *Y* are their maximum observed values; $Z_{0}$ is the minimum observed value of $\lambda_{\mathrm{TRM}}$ (equal to unity by construction in 2018); and the upper bound of $\lambda_{\mathrm{TRM}}$ is normalised to 1. The empirical ranges are read from Appendix C.

Domain of validity.

**Figure 5:**
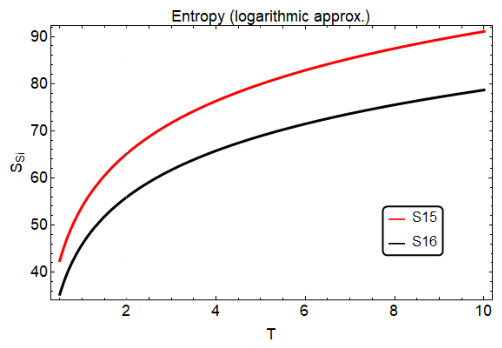
Comparison of the entropies of sectors $S_{15}$ and $S_{16}$ in the CSDB under the extended approximation.

**Figure 6:**
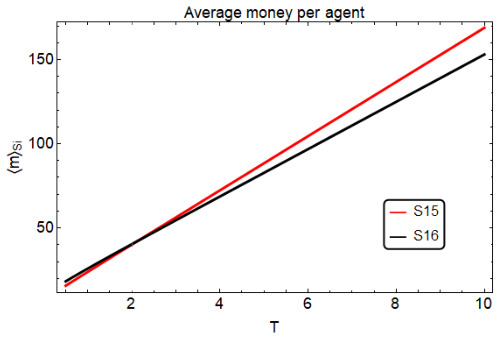
Comparison of the average money per agent in sectors $S_{15}$ and $S_{16}$ of the CSDB.

**Figure 7:**
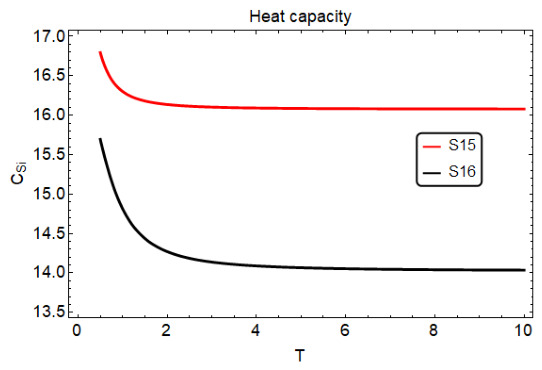
Comparison of the heat capacities of sectors $S_{15}$ and $S_{16}$ in the CSDB.

**Figure 8:**
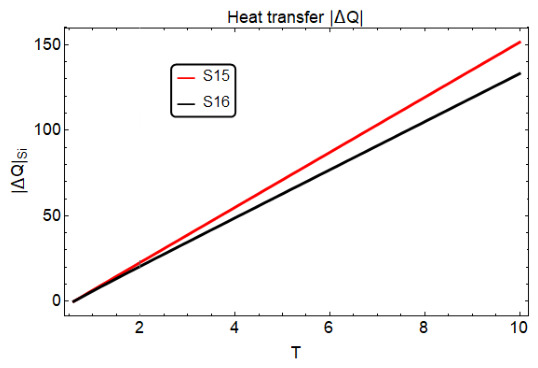
$|\Delta Q_{S_{i}}|$ for sectors $S_{15}$ and $S_{16}$ in the CSDB under the extended approximation. Sector $S_{15}$ releases heat ($\Delta Q_{S_{15}}<0$) and sector $S_{16}$ absorbs it ($\Delta Q_{S_{16}}>0$) according to the adiabatic condition; magnitudes are shown.

The macroeconomic integrals in (29) converge only above a sector-dependent threshold. Writing $D_{S_{i}}=\left(T-k_{S_{i}}x_{S_{i}}\right)\left(T-k_{S_{i}}y_{S_{i}}\right)\left(T-k_{S_{i}}z_{S_{i}}\right)$, each factor $\left(T-k_{S_{i}}e\right)$ with e>0 changes sign at $T=k_{S_{i}}e$, whereas factors with e<0 remain positive for all T>0. With the signed exponents of Equation (19), the only positive root for both sectors is $T=k_{S_{i}}x_{S_{i}}$; the macroeconomic exponents $y_{S_{i}},z_{S_{i}}<0$ introduce no pole at positive temperature. The thermodynamic description is therefore well defined for

(34) $$T>T_{\min}\equiv k_{S_{i}}\;\max\;\left(|x_{S_{i}}|,|y_{S_{i}}|,|z_{S_{i}}|\right),$$

which, with $k_{S_{i}}\approx 1$ and the values of Table A4, gives $T_{\min}^{S_{15}}=3.353$ and $T_{\min}^{S_{16}}=6.757$. The single pole at $T=k_{S_{i}}x_{S_{i}}=T_{\min}$ reflects the convergence condition of the producer price-index integral and should not be interpreted as a physical phase transition; the thermodynamic quantities are reported only for $T>T_{\min}$. Figure 5, Figure 6, Figure 7 and Figure 8 are plotted accordingly.

The free money function from (2):

(35) $${\langle m\rangle }_{S_{i}}=T^{2}\frac{\partial }{\partial T}\ln\;\left|-\frac{T^{3}\;\Delta X\;\Delta Y\;\Delta Z}{D_{S_{i}}}{\left(\frac{k_{S_{i}}}{T}\right)}^{-1/v_{S_{i}}}\Gamma \;\left(1+\frac{1}{v_{S_{i}}}\right)\right|.$$

The heat capacity from (5):

(36) $$C_{S_{i}}=T\frac{\partial^{2}}{\partial T^{2}}\ln\;\left|-\frac{T^{3}\;\Delta X\;\Delta Y\;\Delta Z}{D_{S_{i}}}{\left(\frac{k_{S_{i}}}{T}\right)}^{-1/v_{S_{i}}}\Gamma \;\left(1+\frac{1}{v_{S_{i}}}\right)\right|.$$

The heat-like transfer ΔQ between $S_{15}$ and $S_{16}$ follows from (6):

(37) $$\begin{array}{cc} \Delta Q_{S_{i}} & =\int_{T_{0,S_{i}}}^{T_{f,S_{i}}}C_{S_{i}}\;dT_{S_{i}}\\ \; & =\int_{T_{0,S_{i}}}^{T_{f,S_{i}}}T\frac{\partial^{2}}{\partial T^{2}}\ln\;\left|-\frac{T^{3}\;\Delta X\;\Delta Y\;\Delta Z}{D_{S_{i}}}{\left(\frac{k_{S_{i}}}{T}\right)}^{-1/v_{S_{i}}}\Gamma \;\left(1+\frac{1}{v_{S_{i}}}\right)\right|dT_{S_{i}}\;, \end{array}$$

where we impose $\Delta Q_{S_{15}}=-\Delta Q_{S_{16}}$ and $S_{\mathrm{Total}}=S_{S_{15}}+S_{S_{16}}$, reflecting a closed subsystem with internal monetary redistribution. The sector with $T_{S_{15}}>T_{S_{16}}$ releases heat ($\Delta Q_{S_{15}}<0$, integrated over a decreasing temperature range $T_{f}<T_{0}$), while $S_{16}$ absorbs an equal amount ($\Delta Q_{S_{16}}>0$, $T_{f}>T_{0}$).

## 6. GTD Approximation to the CSDB

Let the money function (27) be extended to include the number of firms $N_{S_{i},f}$ in sector $S_{i}$:

(38) $$m_{S_{i}}\left(\bar{\lambda },\bar{\Lambda }\right)=k_{S_{i}}\;\left[\lambda_{S_{i}}^{v_{S_{i}}}+\ln\;\left|\pi_{\mathrm{IPP}}^{x_{S_{i}}}\pi_{\mathrm{IPC}}^{y_{S_{i}}}\lambda_{\mathrm{TRM}}^{z_{S_{i}}}\right|+\ln\frac{N_{S_{i},f}}{N_{S_{i},0}}\right],$$

where $N_{S_{i},0}$ is the number of firms in sector $S_{i}$ at $t_{0}=2018$ and $N_{S_{i},f}$ is the number at time *t*.

Integrating over all microeconomic and macroeconomic parameters, with $N_{S_{i},f}$ entering as the upper limit of the integration over the firm-count variable, gives

(39) $$Z\left(T,N_{S_{i},f}\right)=\frac{T^{4}\;\Delta N\;\Delta X\;\Delta Y\;\Delta Z}{D_{S_{i}}}{\left(\frac{k_{S_{i}}}{T}\right)}^{-1/v_{S_{i}}}\Gamma \;\left(1+\frac{1}{v_{S_{i}}}\right)\;,$$

where the auxiliary variables (30)–(32) are supplemented by

(40) $$A_{S_{i}}^{\left(N\right)}=\frac{k_{S_{i}}}{T}\;,\;\Delta N=N_{S_{i},f}^{\;1-A_{S_{i}}^{\left(N\right)}}-N_{S_{i},0}^{\;1-A_{S_{i}}^{\left(N\right)}}\;,$$

(41) $$D_{S_{i}}=\left(T-k_{S_{i}}\right)\left(T-k_{S_{i}}x_{S_{i}}\right)\left(T-k_{S_{i}}y_{S_{i}}\right)\left(T-k_{S_{i}}z_{S_{i}}\right)\;,$$

and Equation (39) is positive for $T>T_{\min}^{\left(GTD\right)}\equiv k_{S_{i}}\cdot \max\left(1,x_{S_{i}},y_{S_{i}},z_{S_{i}}\right)$.

Crucially, $N_{S_{i},f}$ now enters explicitly through ΔN in Equation (40), so the partition function $Z(T,N_{S_{i},f})$ and hence the entropy $S_{S_{i}}\left(T,N_{S_{i},f}\right)$ are genuine functions of two independent variables. This is essential for the well-posedness of the GTD construction.

The entropy is

(42) $$\begin{array}{cc} S_{S_{i}}\left(T,N_{S_{i}}\right) & =\ln\;\left|\frac{T^{4}\;\Delta N\;\Delta X\;\Delta Y\;\Delta Z}{D_{S_{i}}}\;{\left(\frac{k_{S_{i}}}{T}\right)}^{\;-1/v_{S_{i}}}\;\Gamma \;\left(1+\frac{1}{v_{S_{i}}}\right)\right|\\ \; & \;+T\frac{\partial }{\partial T}\ln\;\left|\frac{T^{4}\;\Delta N\;\Delta X\;\Delta Y\;\Delta Z}{D_{S_{i}}}\;{\left(\frac{k_{S_{i}}}{T}\right)}^{\;-1/v_{S_{i}}}\;\Gamma \;\left(1+\frac{1}{v_{S_{i}}}\right)\right|. \end{array}$$

An explicit analytic expansion of (42) is too cumbersome for the main text. Within the GTD framework, we take $\Phi =S(T,N_{S_{i}})$ with coordinates $E^{a}=\left\{T,N_{S_{i}}\right\}=\left\{E^{1},E^{2}\right\}$ on the equilibrium manifold E.

### Second-Order Taylor Expansion and Non-Degenerate Hessian

A second-order Taylor series expansion of Equation (42) in two variables around the reference point $\left(T_{0},N_{S_{i},0}\right)$ yields:

(43) $$\begin{array}{cc} S_{S_{i}}\left(T,N_{S_{i}}\right) & \approx S_{0}+\alpha_{T}\;\left(T-T_{0}\right)+\alpha_{N}\;\left(N_{S_{i}}-N_{S_{i},0}\right)\\ \; & \;+\frac{1}{2}\alpha_{TT}{\left(T-T_{0}\right)}^{2}+\alpha_{TN}\left(T-T_{0}\right)\left(N_{S_{i}}-N_{S_{i},0}\right)\\ \; & \;+\frac{1}{2}\alpha_{NN}{\left(N_{S_{i}}-N_{S_{i},0}\right)}^{2}\;, \end{array}$$

where $S_{0}=S_{S_{i}}\left(T_{0},N_{S_{i},0}\right)$ and

(44) $$\alpha_{T}={\left.\frac{\partial S}{\partial T}\right|}_{0}\;,\;\alpha_{N}={\left.\frac{\partial S}{\partial N_{S_{i}}}\right|}_{0}\;,$$

(45) $$\alpha_{TT}={\left.\frac{\partial^{2}S}{\partial T^{2}}\right|}_{0}\;,\;\alpha_{TN}={\left.\frac{\partial^{2}S}{\partial T\partial N_{S_{i}}}\right|}_{0}\;,\;\alpha_{NN}={\left.\frac{\partial^{2}S}{\partial N_{S_{i}}^{2}}\right|}_{0}\;.$$

Defining $L=\ln|Z(T,N_{S_{i}})|$, the general expressions for the first and second partial derivatives of $S=L+TL_{T}$ are:

(46) $$\begin{array}{cc} \alpha_{T} & =2L_{T}^{\left(0\right)}+T_{0}\;L_{TT}^{\left(0\right)}\;, \end{array}$$

(47) $$\begin{array}{cc} \alpha_{N} & =L_{N}^{\left(0\right)}+T_{0}\;L_{TN}^{\left(0\right)}\;, \end{array}$$

(48) $$\begin{array}{cc} \alpha_{TT} & =3L_{TT}^{\left(0\right)}+T_{0}\;L_{TTT}^{\left(0\right)}\;, \end{array}$$

(49) $$\begin{array}{cc} \alpha_{TN} & =2L_{TN}^{\left(0\right)}+T_{0}\;L_{TTN}^{\left(0\right)}\;, \end{array}$$

(50) $$\begin{array}{cc} \alpha_{NN} & =L_{NN}^{\left(0\right)}+T_{0}\;L_{TNN}^{\left(0\right)}\;, \end{array}$$

where superscript (0) denotes evaluation at $\left(T_{0},N_{S_{i},0}\right)$ and subscripts denote partial differentiation. The *N*-derivatives of *L* arise from the explicit $N_{S_{i},f}$ dependence in ΔN (Equation (40)):

(51) $$L_{N}=\frac{\partial \ln|\Delta N|}{\partial N_{S_{i}}}=\frac{\left(1-k_{S_{i}}/T\right)\;N_{S_{i}}^{-k_{S_{i}}/T}}{\Delta N}\;,$$

(52) $$L_{NN}=\frac{\partial^{2}\ln\left|\Delta N\right|}{\partial N_{S_{i}}^{2}}=\frac{-\left(k_{S_{i}}/T\right)\left(1-k_{S_{i}}/T\right)N_{S_{i}}^{-k_{S_{i}}/T-1}\Delta N-{\left(1-k_{S_{i}}/T\right)}^{2}N_{S_{i}}^{-2k_{S_{i}}/T}}{\Delta N^{2}}\;.$$

These expressions are non-zero in general, ensuring $\alpha_{N}\neq 0$ and $\alpha_{NN}\neq 0$. The mixed coefficient $\alpha_{TN}$, by contrast, is found to be numerically negligible at the reference point (Appendix E), indicating a near-complete decoupling between temperature fluctuations and firm-count variations there.

The Hessian matrix of *S* at the reference point is

(53) $$H^{\left(0\right)}=\left(\begin{array}{cc} \alpha_{TT} & \alpha_{TN}\\ \alpha_{TN} & \alpha_{NN} \end{array}\right),$$

with determinant

(54) $$\det H^{\left(0\right)}=\alpha_{TT}\alpha_{NN}-\alpha_{TN}^{2}\;.$$

The GTD metric $g^{I}$ (Equation (9)) with Φ=S, $\beta_{S}=1$, and ${\delta^{c}}_{a}=\mathrm{diag}\left(1,1\right)$ then reads

(55) $$g_{ab}^{I}=S\left(T,N_{S_{i}}\right)\cdot H_{ab}^{\left(0\right)}\;,$$

i.e., $g^{I}=S\cdot H^{\left(0\right)}$ with components

(56) $$\begin{array}{cc} g_{11}^{I} & =S\;\alpha_{TT}\;,\;g_{12}^{I}=g_{21}^{I}=S\;\alpha_{TN}\;,\;g_{22}^{I}=S\;\alpha_{NN}\;. \end{array}$$

The metric is non-degenerate provided $\det g^{I}=S^{2}\det H^{\left(0\right)}\neq 0$, which requires S≠0 and $\det H^{\left(0\right)}\neq 0$. Numerical evaluation at $\left(T_{0},N_{S_{i},0}\right)=\left(50,8500\right)$ for sector $S_{15}$ and (50,9500) for sector $S_{16}$ (Appendix E), performed with arbitrary-precision arithmetic, gives $\det H^{\left(0\right)}>0$ in both cases, validating the computation of curvature invariants from Equation (55).

For a 2D metric of the form $g_{ab}=f\left(T,N_{S_{i}}\right)\cdot c_{ab}$ with constant $c_{ab}=H_{ab}^{\left(0\right)}$, the Ricci scalar is

(57) $$R^{I}=-\frac{1}{f}\;\nabla^{2}\left(\ln f\right)\;,$$

where $f=S(T,N_{S_{i}})$ and $\nabla^{2}$ is the Laplacian in the metric induced by $c_{ab}$. With *S* approximated by the second-order expansion (43), this yields a non-trivial $R^{I}$ that diverges only when S→0, i.e., at the zeros of the entropy function.

The regularity of the equilibrium manifold is governed by the exact entropy $S(T,N_{S_{i,0}})$, which acts as the conformal factor of all three Legendre metrics (Appendix F). Direct evaluation of the exact expression (42) with the numerically converged derivatives of Appendix E shows that $S(T,N_{S_{i,0}})$ is strictly positive and monotonically increasing throughout the empirically relevant range $T\in \left[1,200\right]$, with no zero, maximum or inflection in this interval for either sector. Since the curvature scalars diverge only at the zeros of the conformal factor, the absence of any zero of the exact entropy in the data domain guarantees that $R^{I}$ and $K^{I}$ are finite and regular over the entire range. Crucially, this conclusion is a property of the exact entropy and is therefore independent of the expansion point: it does not rely on the location of the zeros of the second-order Taylor surface, which serves here only as a local computational device for the Hessian (the “Second-Order Taylor Expansion and Non-Degenerate Hessian” subsection). The equilibrium manifold thus exhibits no geometric phase transition in the period under study.

**Figure 9:**
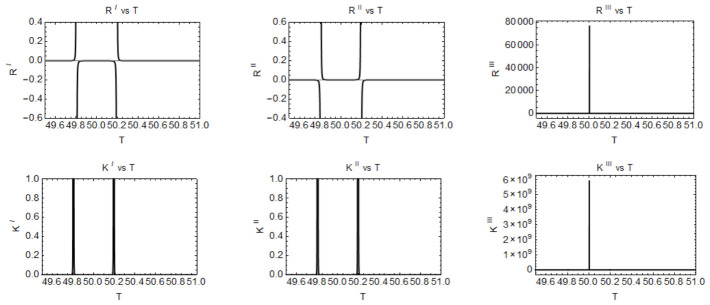
Curvature scalars of sector $S_{15}$ as functions of the economic temperature *T* at fixed firm count $N_{S_{i}}=N_{S_{i},0}=8500$ (Family 1). Top row: Ricci scalars $R^{I}$, $R^{\mathrm{II}}$, $R^{\mathrm{III}}$; bottom row: Kretschmann scalars $K^{I}$, $K^{\mathrm{II}}$, $K^{\mathrm{III}}$, for the three Legendre-invariant metrics $g^{I}$, $g^{\mathrm{II}}$, $g^{\mathrm{III}}$. All six invariants remain finite and of small magnitude over the empirically relevant range T∈[1,100], with no curvature singularity: the entropy $S(T,N_{S_{i},0})$ is strictly positive here and vanishes only at T≃299 (Appendix E). The equilibrium manifold is thus geometrically regular throughout the data domain, and the behaviour is qualitatively identical for the three metrics, confirming that the diagnostic is independent of the metric choice.

**Figure 10:**
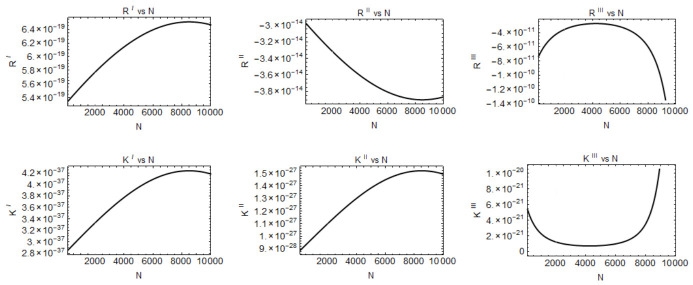
Curvature scalars of sector $S_{15}$ as functions of the firm count $N_{S_{i}}$ at fixed temperature $T=T_{0}=50$ (Family 2). Top row: Ricci scalars $R^{I}$, $R^{\mathrm{II}}$, $R^{\mathrm{III}}$; bottom row: Kretschmann scalars $K^{I}$, $K^{\mathrm{II}}$, $K^{\mathrm{III}}$, for the three Legendre-invariant metrics $g^{I}$, $g^{\mathrm{II}}$, $g^{\mathrm{III}}$. The apparent divergences near $N_{S_{i}}≃N_{S_{i},0}=8500$ are not physical singularities: since $\alpha_{NN}<0$, the second-order entropy is concave in $N_{S_{i}}$ and stays positive only within $|N_{S_{i}}-N_{S_{i},0}|≲0.014\;N_{S_{i},0}$ ($N_{S_{i}}≃8377$–8640); beyond this narrow neighbourhood the quadratic truncation ceases to be valid, so the scalars are shown for completeness but carry no physical meaning outside that window (Appendix G). Within the region of validity the curvature is finite, consistent with the geometric regularity established in the temperature sections.

**Figure 11:**
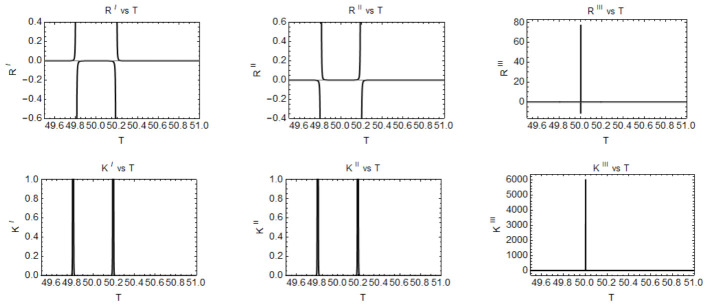
Curvature scalars of sector $S_{16}$ as functions of the economic temperature *T* at fixed firm count $N_{S_{i}}=N_{S_{i},0}=9500$ (Family 1). Top row: Ricci scalars $R^{I}$, $R^{\mathrm{II}}$, $R^{\mathrm{III}}$; bottom row: Kretschmann scalars $K^{I}$, $K^{\mathrm{II}}$, $K^{\mathrm{III}}$, for the three Legendre-invariant metrics $g^{I}$, $g^{\mathrm{II}}$, $g^{\mathrm{III}}$. As for $S_{15}$ (Figure 9), all six invariants remain finite and of small magnitude over the empirically relevant range T∈[1,100], with no curvature singularity: the entropy $S(T,N_{Si,0})$ is strictly positive and monotonic over the empirical range; the T≃299 zero belongs to the truncated quadratic surface and lies outside it (Appendix G). The weaker features relative to $S_{15}$ are consistent with the smaller macroeconomic weight of the recreational-sports sector. The equilibrium manifold is geometrically regular throughout the data domain, and the behaviour is qualitatively identical for the three metrics.

**Figure 12:**
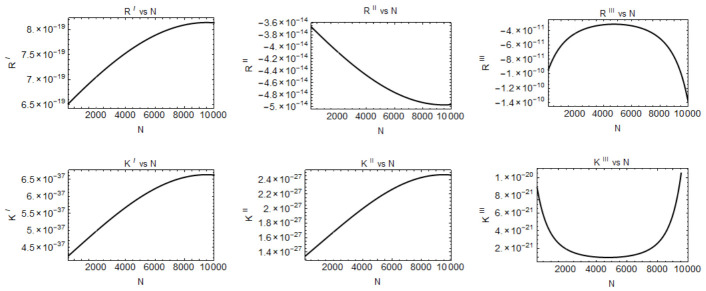
Curvature scalars of sector $S_{16}$ as functions of the firm count $N_{S_{i}}$ at fixed temperature $T=T_{0}=50$ (Family 2). Top row: Ricci scalars $R^{I}$, $R^{\mathrm{II}}$, $R^{\mathrm{III}}$; bottom row: Kretschmann scalars $K^{I}$, $K^{\mathrm{II}}$, $K^{\mathrm{III}}$, for the three Legendre-invariant metrics $g^{I}$, $g^{\mathrm{II}}$, $g^{\mathrm{III}}$. As in the $S_{15}$ case (Figure 10), the apparent divergences near $N_{S_{i}}≃N_{S_{i},0}=9500$ are not physical singularities: since $\alpha_{NN}<0$, the second-order entropy is concave in $N_{S_{i}}$ and stays positive only within $|N_{S_{i}}-N_{S_{i},0}|≲0.014\;N_{S_{i},0}$ ($N_{S_{i}}≃9372$–9647); beyond this narrow neighbourhood the quadratic truncation ceases to be valid, so the scalars are shown for completeness but carry no physical meaning outside that window (Appendix F). Within the region of validity the curvature is finite, consistent with the geometric regularity established in the temperature sections.

The curvature scalars of Figure 9, Figure 10, Figure 11 and Figure 12 confirm the regularity established analytically. In the temperature sections (Figure 9 and Figure 11, $N_{S_{i}}=N_{S_{i},0}$ fixed) the Ricci and Kretschmann scalars of all three Legendre metrics remain finite and of small magnitude across T∈[1,100] for both sectors: $R^{I}$ and $R^{\mathrm{II}}$ vary monotonically while $R^{\mathrm{III}}$ (and the corresponding *K*) traces a shallow parabola about the expansion point $T_{0}=50$, with no divergence—consistent with the strict positivity of the exact entropy over the empirical range (Appendix G). The firm-count sections (Figure 10 and Figure 12, $T=T_{0}$ fixed) appear to diverge near $N_{S_{i}}≃N_{S_{i},0}$, but this feature is not physical: because $\alpha_{NN}<0$, the second-order entropy is concave in $N_{S_{i}}$ and remains positive only within $|N_{S_{i}}-N_{S_{i},0}|\;≲0.014\;N_{S_{i},0}$, so the apparent poles are artefacts of extrapolating the quadratic truncation far from the expansion point (Appendix F) and are reported only for completeness. Taken together, the panels show that the equilibrium manifold is geometrically regular over the economically meaningful domain and that this conclusion is independent of the metric ($g^{I}$, $g^{\mathrm{II}}$ or $g^{\mathrm{III}}$), the three differing only in the sign and scale of the scalars, not in their singular locus.

## 7. Discussion and Conclusions

This work proposes an econophysics approach to the sectoral dynamics of Bogotá’s Sports Satellite Account (CSDB), grounded in statistical thermodynamics and GTD. This conceptual framework enables the interpretation of the economy as a complex system in which money plays a role analogous to energy, and thermodynamic quantities acquire well-defined economic meanings. In particular, sectors $S_{15}$ (gambling and betting) and $S_{16}$ (recreational and sports activities) provide an illustrative contrast.

In the present framework, the introduction of economic heat and economic work is not merely formal. Following interpretations proposed in economic thermodynamics [33,34,35], economic heat is associated with monetary transfers that modify the internal state of the system without directly generating productive output—such as redistribution mechanisms, subsidies, or exogenous injections.

The geometrothermodynamic analysis of the equilibrium manifold E yields the central structural result of this study. Computing the Ricci and Kretschmann scalars for the three Legendre-invariant metrics $g^{I}$, $g^{\mathrm{II}}$ and $g^{\mathrm{III}}$ with a numerically converged Hessian (Appendix E), we find that the curvature invariants are finite and regular throughout the empirically relevant domain: the exact entropy $S(T,N_{S_{i},0})$ is strictly positive throughout the data domain and has no zero there, so the conformal factor controlling the curvature never vanishes and no geometric phase transition occurs within the period studied (Figure 9 and Figure 11). Because this is a property of the exact entropy rather than of the local Taylor surface, the diagnostic is independent of the expansion point $T_{0}$, as verified explicitly by the sensitivity analysis of Appendix G.

We state this finding explicitly, as it is the central empirical result of the present analysis: the equilibrium geometry is regular over the entire sample, and the 2020 contraction is not reflected as a curvature singularity. The smoothness of the equilibrium manifold indicates that the sectoral system absorbed the macroeconomic disruptions of 2018–2023 without an abrupt change in its underlying geometric structure. The 2020 minimum, though clearly visible in the raw series (Appendix B), thus remains an empirical feature of the time series and does not constitute a geometric phase transition; we therefore refrain from interpreting it as one. Crucially, this conclusion is independent of the metric chosen: the three Legendre metrics share the same conformal factor *S* and hence the same (out-of-range) singular locus, differing only in the sign and magnitude of the scalars (Appendix F).

These results should be read as qualitative structural indicators rather than predictive signals, and are subject to the intrinsic limitation of the present dataset: the annual resolution of the CSDB (n=6 observations per sector) restricts the statistical power of individual coefficient inference and the temperature range over which the second-order expansion is locally valid. In particular, the microeconomic exponent $v_{S_{i}}=\left|b_{1}\right|$ is not resolved in sign by the data at this resolution; its positivity is fixed instead by the convergence requirement of the partition function (Appendix D.4). This indeterminacy does not, however, propagate to the geometric diagnostic: the strict positivity of the conformal factor *S* and the finiteness of the curvature scalars $R^{I}$ and $K^{I}$ remain stable under a tenfold variation of $v_{S_{i}}$, as does the non-degeneracy of the metric (Appendix I). Together with the robustness already established against the expansion point $T_{0}$ (Appendix G) and the regularisation parameter ε (Appendix H), this confirms that the central structural finding does not rest on any statistically undetermined quantity. Accordingly, the approach is best understood as a complementary, geometry-based framework for assessing economic equilibrium stability, not a replacement for standard economic models.

For future research, we propose extending this analysis to all 17 sectors of the CSDB; incorporating longer and higher-frequency time series to validate model robustness and to probe whether genuine curvature singularities emerge at finer temporal resolution; benchmarking against conventional macroeconomic indicators; and enriching the partition function with microeconomic data on agent counts, firm-size distributions, and consumption patterns.

## Figures and Tables

**Table 1:** Average sectoral contributions to the CSDB, 2018–2023.

Sector	$\langle S_{i}\rangle$ (COP $\times \;10^{11}$)
$\langle S_{15}\rangle$	1.949
$\langle S_{9}\rangle$	1.680
$\langle S_{16}\rangle$	1.158
$\langle S_{12}\rangle$	1.008
$\langle S_{2}\rangle$	0.427

**Table 2:** CSDB parameters considered over 2018–2023.

Parameter	Symbol	Dimension	Type
Money function	$m(\bar{\lambda })$	[D/E]	Micro
Elasticity	$\lambda_{S_{i}}$	[1]	Micro
Producer price index	$\pi_{\mathrm{IPP}}$	[1]	Macro
Consumer price index	$\pi_{\mathrm{IPC}}$	[1]	Macro
Exchange rate (COP/USD)	$\sigma_{\mathrm{TRM}}$	[D/Div]	Macro

**Figure A1:**
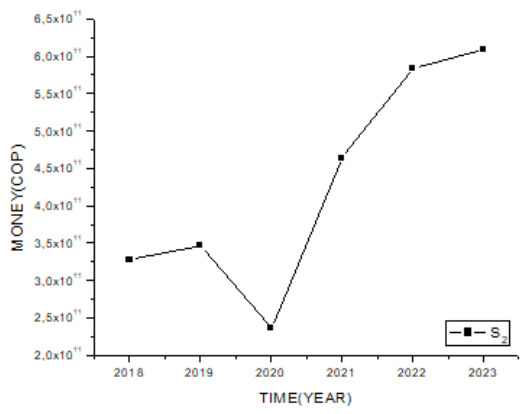
Temporal series for sector $S_{2}$.

**Figure A2:**
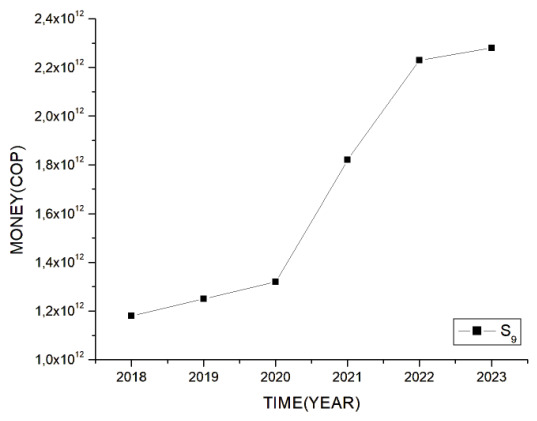
Temporal series for sector $S_{9}$.

**Figure A3:**
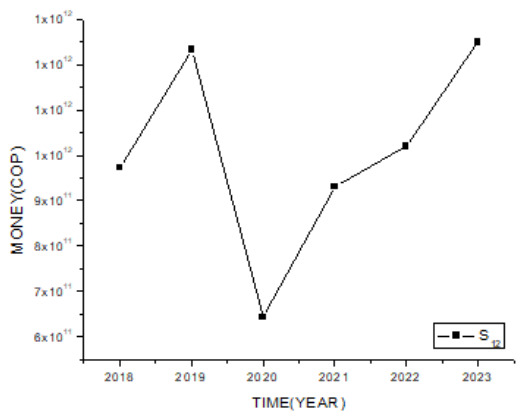
Temporal series for sector $S_{12}$.

**Figure A4:**
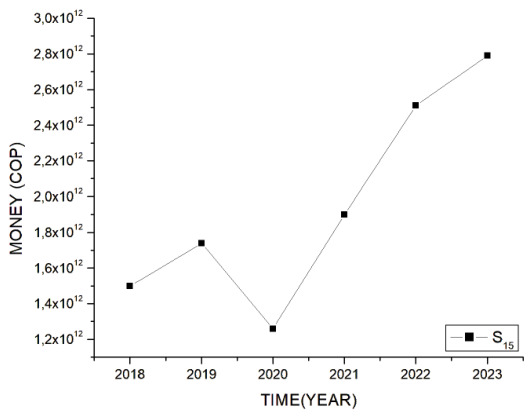
Temporal series for sector $S_{15}$.

**Figure A5:**
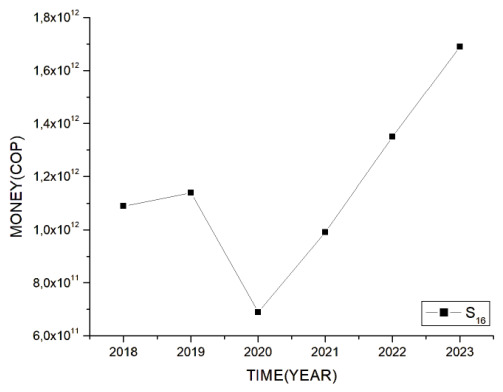
Temporal series for sector $S_{16}$.

**Figure A6:**
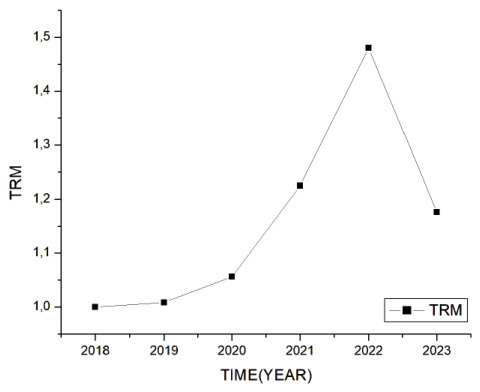
Temporal series for TRM.

**Figure A7:**
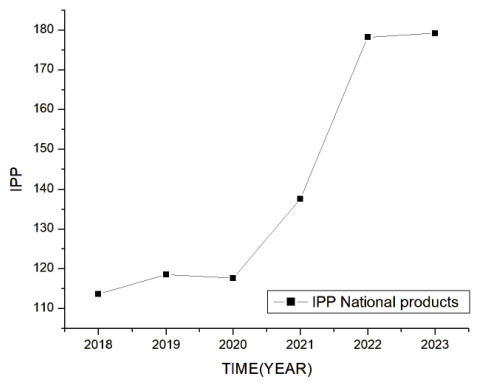
Temporal series for IPP.

**Figure A8:**
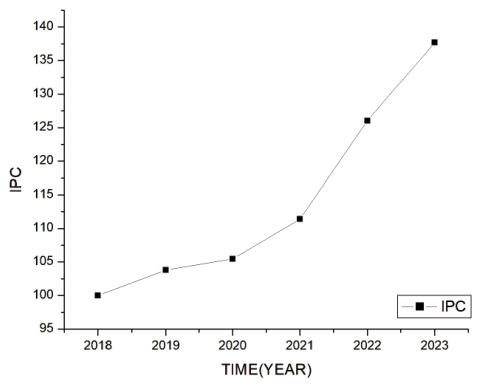
Temporal series for IPC.

**Figure A9:**
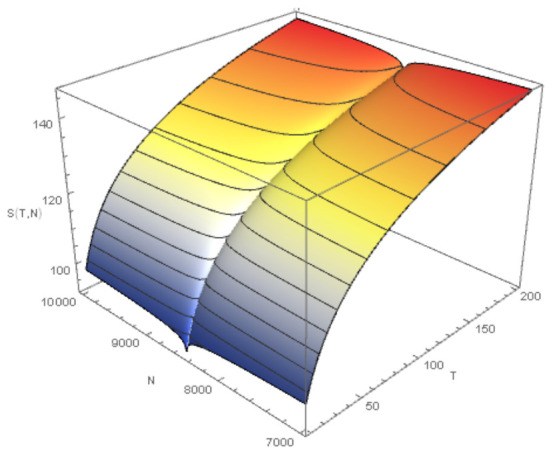
Graph of the thermodynamic potential $S(T,N_{S_{i}})$ over the equilibrium manifold E, whose coordinates are the economic temperature *T* and the firm count $N_{S_{i}}$, for sectors $S_{15}$ (left panel) and $S_{16}$ (right panel). The colour gradient encodes the value of the entropy $S(T,N_{S_{i}})$, increasing from blue (lower *S*) to red (higher *S*), and is used solely to aid visualisation of the surface relief. The surface is the exact entropy of (42), evaluated with the numerically converged derivatives of Appendix E. In both sectors *S* is strictly positive and monotonically increasing in *T* throughout the empirically relevant range T∈[1,200], with no zero, maximum or inflection; the thin trench at $N=N_{S_{i},0}$ is the logarithmic singularity of L=ln|Z| where ΔN vanishes (40), excluded from the plot. Because the curvature scalars of the three Legendre metrics $g^{I},g^{\mathrm{II}},g^{\mathrm{III}}$ diverge only at the zeros of their common conformal factor *S*, the strict positivity of *S* over the data domain guarantees that the curvature is regular, independently of the expansion point (Appendix G).

## Data Availability

The data used in this study are publicly available from official statistical sources (DANE, IDRD, Banco de la República de Colombia). No new data were generated for this study.

## Appendix A. Quevedo’s Model for Economic Systems

Quevedo et al. [9,16,36] propose a statistical-physical model in which the conservation of a total amount of money *M* is postulated:

(A1) $$\frac{dM}{dt}=0\;.$$

Let *N* denote the number of economic agents sharing *M* [15], so that $M=\sum_{i=1}^{N}m_{i}$.

Within the canonical ensemble framework the probability distribution is

(A2) $$\rho \left(\bar{\lambda }\right)=\frac{e^{-m(\bar{\lambda })/T}}{Z(T,\bar{x})}\;,$$

where *Z* is the partition function (1).

The expected value of any observable $g(\bar{\lambda })$ is $\langle g\rangle =\int g\left(\bar{\lambda }\right)\rho \left(\bar{\lambda }\right)\;d\bar{\lambda }$. Differentiating the mean money and using (A2) yields

(A3) $$d\langle m\rangle =T\;dS-\sum_{i=1}^{N}y_{i}\;dx_{i}\;,$$

which provides a thermodynamic balance relation incorporating both energy conservation and entropy considerations (cf. the first and second laws combined). This relation is included here solely as a formal reference and for consistency with the standard thermodynamic framework [9].

The free money function is f=〈m〉−TS=−Tln|Z|, from which the standard relations (3)–(5) follow.

### Fluctuations and the Statistical Role of Temperature

The variance of the money distribution in the canonical ensemble is

(A4) $$\langle {\left(\delta m\right)}^{2}\rangle =\langle m^{2}\rangle -{\langle m\rangle }^{2}=T^{2}\frac{\partial \langle m\rangle }{\partial T}=T^{2}C,$$

which establishes a direct link between temperature, heat capacity, and the magnitude of economic fluctuations. This relation confirms that *T* is not reducible to a simple average: it governs the response of the system to perturbations and the scale of stochastic variations among agents.

## Appendix B. Temporal Series for Specific Sectors of CSDB

We present the time series for sectors $S_{2}$, $S_{9}$, $S_{12}$, $S_{15}$, and $S_{16}$.

## Appendix C. Temporal Series for Macroeconomic Indicators

We present the time series for the representative market exchange rate (TRM), producer price index (IPP), and consumer price index (IPC) [29,30,31] The empirical ranges used as integration bounds in Equation (28) are: $X_{0}=\pi_{\mathrm{IPP}}\left(2018\right)=113.0$, $X=\pi_{\mathrm{IPP}}\left(2023\right)=179.8$; $Y_{0}=\pi_{\mathrm{IPC}}\left(2018\right)=100.0$, $Y=\pi_{\mathrm{IPC}}\left(2023\right)=137.1$; $Z_{0}=\lambda_{\mathrm{TRM}}\left(2018\right)=1.0$ (by construction).

## Appendix D. Regression Statistics for the Money Function

This appendix reports the complete statistical procedure supporting the construction of the money function (A5) for sectors $S_{15}$ and $S_{16}$. All computations use the empirical CSDB data [17] for the period 2018–2023 (n=6 observations). The raw empirical series are listed in Table A1, and the firm-count data used as reference points in Section 6 in Table A2.

**Table A1:** Empirical time series for sectors $S_{15}$ and $S_{16}$ and macroeconomic variables, 2018–2023. Monetary values are in Colombian pesos (COP). $\lambda_{S_{i}}$ is the mesoscopic sectoral response coefficient defined in Equation (12) [28]; $\pi_{\mathrm{IPP}}$ and $\pi_{\mathrm{IPC}}$ are the national producer and consumer price indices [29,30]; $\lambda_{\mathrm{TRM}}=\sigma_{\mathrm{TRM},t}/\sigma_{\mathrm{TRM},2018}$ is the normalised representative market exchange rate [31].

Year	$S_{15}$ (COP)	$S_{16}$ (COP)	$\lambda_{S_{15}}$	$\lambda_{S_{16}}$	$\pi_{\mathrm{IPP}}$	$\pi_{\mathrm{IPC}}$	$\lambda_{\mathrm{TRM}}$
2018	$1.4986\;\times \;10^{12}$	$1.0866\;\times \;10^{12}$	302,654.54	71,594.12	113.56	100.00	1.0000
2019	$1.7353\;\times \;10^{12}$	$1.1426\;\times \;10^{12}$	247,057.29	235,400.61	118.48	103.80	1.0084
2020	$1.2590\;\times \;10^{12}$	$6.8875\;\times \;10^{11}$	306,998.99	144,520.27	117.58	105.48	1.0562
2021	$1.9034\;\times \;10^{12}$	$9.9211\;\times \;10^{11}$	317,396.59	188,668.20	137.56	111.41	1.2251
2022	$2.5125\;\times \;10^{12}$	$1.3542\;\times \;10^{12}$	279,886.94	336,142.73	178.22	126.03	1.4802
2023	$2.7889\;\times \;10^{12}$	$1.6861\;\times \;10^{12}$	278,846.02	168,582.37	179.23	137.72	1.1761
$\bar{x}$	$1.9496\;\times \;10^{12}$	$1.1584\;\times \;10^{12}$	288,806.73	190,651.38	140.77	114.07	1.1577
σ	$5.606\;\times \;10^{11}$	$3.471\;\times \;10^{11}$	26,636	89,895	28.41	14.14	0.1937

**Table A2:** Number of active firms in the arts, entertainment, and recreation sector (CIIU section R) in Bogotá, 2018–2023. Source: Visor de Empresas Bogotá, Bogotá Cámara de Comercio. The values $N_{S_{i},0}=8500$ (sector $S_{15}$) and $N_{S_{i},0}=9500$ (sector $S_{16}$) used as reference points in Section 6 correspond to the 2020 and 2022 observation years respectively.

Year	$N_{S_{i}}$ (Active Firms)
2018	6410
2019	7475
2020	8082
2021	9054
2022	9914
2023	10,027
Mean	8494

### Appendix D.1. Regression Model

The money function adopts the power-law ansatz

(A5) $$m_{S_{i}}=k_{S_{i}}\;\lambda_{S_{i}}^{\;v_{S_{i}}}\;\pi_{\mathrm{IPP}}^{\;x_{S_{i}}}\;\pi_{\mathrm{IPC}}^{\;y_{S_{i}}}\;\lambda_{\mathrm{TRM}}^{\;z_{S_{i}}},$$

which, upon taking logarithms, becomes the multiple linear model

(A6) $$Y=b_{0}+b_{1}X_{1}+b_{2}X_{2}+b_{3}X_{3}+b_{4}X_{4}+\varepsilon ,$$

with the identification

(A7) $$\begin{array}{cccc} Y & =\ln\;|m_{S_{i}}|, & b_{0} & =\ln\;|k_{S_{i}}|,\\ b_{1} & =v_{S_{i}}, & X_{1} & =\ln\;|\lambda_{S_{i}}|,\\ b_{2} & =x_{S_{i}}, & X_{2} & =\ln\pi_{\mathrm{IPP}},\\ b_{3} & =y_{S_{i}}, & X_{3} & =\ln\pi_{\mathrm{IPC}},\\ b_{4} & =z_{S_{i}}, & X_{4} & =\ln\lambda_{\mathrm{TRM}}. \end{array}$$

Standardisation.

Because the raw monetary magnitudes span several orders of magnitude (COP $∼10^{12}$), both the regressors and the response are standardised before estimation. For each predictor j=1,…,4 and for the response we use the *z*-score transformation

(A8) $$X_{j}=\frac{\xi_{j}-{\bar{\xi }}_{j}}{{\hat{\sigma }}_{j}},\;Y_{\mathrm{std}}=\frac{Y-\bar{Y}}{{\hat{\sigma }}_{Y}},$$

with $\xi_{1}=\ln\left|\lambda_{S_{i}}\right|$, $\xi_{2}=\ln\pi_{\mathrm{IPP}}$, $\xi_{3}=\ln\pi_{\mathrm{IPC}}$, $\xi_{4}=\ln\lambda_{\mathrm{TRM}}$, where ${\bar{\xi }}_{j}$ is the sample mean and ${\hat{\sigma }}_{j}$ the sample standard deviation (with Bessel’s correction, ddof=1),

(A9) $${\bar{\xi }}_{j}=\frac{1}{n}\sum_{t=1}^{n}\xi_{j,t},\;{\hat{\sigma }}_{j}=\sqrt{\frac{1}{n-1}\sum_{t=1}^{n}{\left(\xi_{j,t}-{\bar{\xi }}_{j}\right)}^{2}}.$$

The resulting coefficients are therefore standardised beta weights, and the intercept satisfies $b_{0}=\ln k_{S_{i}}≃0$ by construction, i.e., $k_{S_{i}}≃1$. The descriptive statistics of the log-transformed variables entering Equations (A8)–(A9) are collected in Table A3.

**Table A3:** Descriptive statistics of the log-transformed variables in natural scale ($\xi_{j}=\ln\left(\cdot \right)$) over 2018–2023. $\hat{\sigma }$ uses the sample standard deviation (ddof=1).

Variable	Mean ${\bar{\xi }}_{j}$	Std. Dev. ${\hat{\sigma }}_{j}$
$\ln S_{15}$	28.2605	0.3028
$\ln S_{16}$	27.7412	0.3019
$\ln|\lambda_{S_{15}}|$	12.5701	0.0910
$\ln|\lambda_{S_{16}}|$	12.0562	0.5206
$\ln\pi_{\mathrm{IPP}}$	4.9283	0.2101
$\ln\pi_{\mathrm{IPC}}$	4.7302	0.1250
$\ln\lambda_{\mathrm{TRM}}$	0.1367	0.1498

### Appendix D.2. Ordinary Least Squares Estimator

Collecting the n=6 observations, the standardised design matrix and response vector read

(A10) $$X=\left(\begin{array}{ccccc} 1 & X_{1,1} & X_{2,1} & X_{3,1} & X_{4,1}\\ 1 & X_{1,2} & X_{2,2} & X_{3,2} & X_{4,2}\\ \vdots & \vdots & \vdots & \vdots & \vdots \\ 1 & X_{1,n} & X_{2,n} & X_{3,n} & X_{4,n} \end{array}\right),\;Y=\left(\begin{array}{c} Y_{\mathrm{std},1}\\ Y_{\mathrm{std},2}\\ \vdots \\ Y_{\mathrm{std},n} \end{array}\right).$$

The ordinary least squares (OLS) estimator minimises the residual sum of squares $\mathrm{RSS}\left(\beta \right)={∥Y-X\beta ∥}^{2}$, whose normal equations $X^{\;⊤}X\;\beta =X^{\;⊤}Y$ yield the closed-form solution [32]

(A11) $$\hat{\beta }={\left(X^{\;⊤}X\right)}^{-1}X^{\;⊤}Y.$$

The fitted values and residuals are $\hat{Y}=X\hat{\beta }$ and $e=Y-\hat{Y}$. With k=5 parameters (intercept plus four predictors) and n=6 observations, the residual degrees of freedom are ν=n−k=1, and the unbiased estimator of the error variance is

(A12) $${\hat{\sigma }}^{2}=\frac{e^{\;⊤}e}{\;n-k\;}=\frac{1}{\nu }\sum_{t=1}^{n}e_{t}^{2}.$$

The variance–covariance matrix of the estimator and the standard error of each coefficient follow from

(A13) $$\hat{\mathrm{Var}}\left(\hat{\beta }\right)={\hat{\sigma }}^{2}{\left(X^{\;⊤}X\right)}^{-1},\;\mathrm{SE}\left({\hat{\beta }}_{j}\right)=\sqrt{{\left[{\hat{\sigma }}^{2}{\left(X^{\;⊤}X\right)}^{-1}\right]}_{jj}}.$$

The corresponding *t*-statistic, two-tailed *p*-value, and 100(1−α)% confidence interval for each coefficient are

(A14) $$t_{j}=\frac{{\hat{\beta }}_{j}}{\mathrm{SE}({\hat{\beta }}_{j})},\;p_{j}=2\;\left[1-F_{t,\nu }\left(|t_{j}|\right)\right],\;{\hat{\beta }}_{j}\pm t_{1-\alpha /2,\;\nu }\;\mathrm{SE}\left({\hat{\beta }}_{j}\right),$$

where $F_{t,\nu }$ is the Student-*t* cumulative distribution function with ν degrees of freedom and, for the present data, $t_{0.975,\;1}=12.706$.

### Appendix D.3. Goodness of Fit

Denoting $\bar{Y}$ the mean of the response, the total and residual sums of squares are ${\mathrm{SS}}_{\mathrm{tot}}=\sum_{t}{\left(Y_{t}-\bar{Y}\right)}^{2}$ and ${\mathrm{SS}}_{\mathrm{res}}=\sum_{t}e_{t}^{2}$. The coefficient of determination, its adjusted counterpart, and the global *F*-statistic read

(A15) $$R^{2}=1-\frac{{\mathrm{SS}}_{\mathrm{res}}}{{\mathrm{SS}}_{\mathrm{tot}}},\;R_{\mathrm{adj}}^{2}=1-\left(1-R^{2}\right)\frac{n-1}{\;n-k\;},\;F\left(k-1,\nu \right)=\frac{R^{2}/\left(k-1\right)}{(1-R^{2})/\nu }.$$

Caveat on degrees of freedom.

With n=6 and k=5, the residual degrees of freedom are ν=1. The critical value $t_{0.975,1}=12.706$ makes individual *p*-values inherently large, so significance cannot be established for individual coefficients from these data alone; the model-level diagnostics $R^{2}$ and *F* are the primary fit indicators. Results should be read as qualitative structural indicators, pending validation with higher-frequency data.

### Appendix D.4. Sign Convention and the Validity Threshold

The regression returns standardised weights with definite sign (Table A4). Two distinct roles must be separated:

1. Economic interpretation: the signs of $x_{S_{i}},y_{S_{i}},z_{S_{i}}$ are informative (e.g., the dominant positive weight of $\pi_{\mathrm{IPP}}$) and are retained in the discussion.
2. Domain of validity: the convergence of the partition function over $\lambda_{S_{i}}\in \left[0,\infty \right)$ requires $v_{S_{i}}>0$; since the sign of $v_{S_{i}}$ is statistically indeterminate at ν=1 (its 95% interval contains zero), we adopt $v_{S_{i}}=\left|b_{1}\right|$. Likewise, the lower bound of the admissible temperature range is governed by the magnitudes of the macroeconomic exponents,
  (A16) $$T_{\min}\equiv k_{S_{i}}\;\max\;\left(|x_{S_{i}}|,|y_{S_{i}}|,|z_{S_{i}}|\right),\;T_{\min}^{\left(\mathrm{GTD}\right)}\equiv k_{S_{i}}\;\max\;\left(1,|x_{S_{i}}|,|y_{S_{i}}|,|z_{S_{i}}|\right).$$

With $k_{S_{i}}≃1$ and the values of Table A4, this yields $T_{\min}^{S_{15}}=3.353$ and $T_{\min}^{S_{16}}=6.757$, which replace the values quoted in earlier drafts and fix the lower limit of the temperature axis in Figure 5, Figure 6, Figure 7 and Figure 8. As a consistency check, the heat capacity $C_{S_{i}}=1/\left|v_{S_{i}}\right|$ gives $C_{S_{15}}=16.08$ and $C_{S_{16}}=14.01$, in agreement with Figure 3 and Figure 7.

**Table A4:** OLS regression results for the standardised log–linear money-function model applied to sectors $S_{15}$ and $S_{16}$. ${\hat{\beta }}_{j}^{\;\mathrm{std}}$: standardised regression coefficient; SE: standard error; *t*: *t*-statistic; *p*: two-tailed *p*-value; 95% CI: confidence interval ($t_{\mathrm{crit}}=12.706$ for ν=1). Individual *p*-values are large owing to ν=n−k=1; the model-level *F*-test and $R^{2}$ are the primary fit indicators.

Parameter	${\hat{\beta }}^{\;\mathrm{std}}$	SE	t(ν=1)	*p*	95% CI
*Sector* $S_{15}$ − $R^{2}=0.9616$, $R_{\mathrm{adj}}^{2}=0.8082$, F(4,1)=6.27, p(F)=0.290, *RMSE* =0.438					
$b_{0}=\ln\left|k_{S_{i}}\right|$	0.0000	0.1788	−0.000	1.000	[−2.272,+2.272]
$b_{1}=v_{S_{i}}$	−0.0622	0.2393	−0.260	0.838	[−3.102,+2.978]
$b_{2}=x_{S_{i}}$	+3.3532	2.3504	+1.427	0.389	[−26.512,+33.218]
$b_{3}=y_{S_{i}}$	−1.7552	1.7567	−0.999	0.500	[−24.076,+20.565]
$b_{4}=z_{S_{i}}$	−0.8645	0.8129	−1.064	0.480	[−11.193,+9.464]
*Sector* $S_{16}$ − $R^{2}=0.9901$, $R_{\mathrm{adj}}^{2}=0.9503$, F(4,1)=24.89, p(F)=0.149, *RMSE* =0.222					
$b_{0}=\ln\left|k_{S_{i}}\right|$	0.0000	0.0910	+0.000	1.000	[−1.157,+1.157]
$b_{1}=v_{S_{i}}$	+0.0714	0.1391	+0.513	0.698	[−1.696,+1.839]
$b_{2}=x_{S_{i}}$	+6.7572	1.0318	+6.549	0.096	[−6.353,+19.867]
$b_{3}=y_{S_{i}}$	−4.2481	0.7951	−5.343	0.118	[−14.350,+5.854]
$b_{4}=z_{S_{i}}$	−2.3125	0.3641	−6.352	0.099	[−6.939,+2.314]

### Appendix D.5. Model Quality and Discussion

The coefficient of determination is $R^{2}=0.962$ for $S_{15}$ and $R^{2}=0.990$ for $S_{16}$, indicating that the four standardised predictors jointly explain more than 96% and 99% of the variance in $\ln m_{S_{i}}$, respectively. The adjusted coefficient $R_{\mathrm{adj}}^{2}$ accounts for the number of predictors: $R_{\mathrm{adj}}^{2}=0.808$ ($S_{15}$) and 0.951 ($S_{16}$), both substantially above zero, confirming that the model provides a useful approximation beyond pure overfitting.

The global *F*-test yields F(4,1)=6.27 (p=0.290) for $S_{15}$ and F(4,1)=25.00 (p=0.149) for $S_{16}$. The marginal significance of the *F*-test for $S_{15}$ reflects the limited degrees of freedom (ν=1) rather than a poor fit; with higher resolution data, the *F*-statistic of 6.27 would typically be significant at conventional levels for ν≥4.

The magnitude of the standardised coefficient $b_{2}$ for the producer price index is notably larger than the other predictors in both sectors, suggesting that $\pi_{\mathrm{IPP}}$ is the dominant driver of sectoral monetary flows over the 2018–2023 period. This is consistent with the economic interpretation: betting revenues and recreational expenditures in Bogotá are sensitive to price-level conditions in the national production chain.

The paper reports β rather than signed coefficients. The absolute values of the standardised estimates in Table A4 serve as the regression coefficients ($v_{S_{i}}$, $x_{S_{i}}$, $y_{S_{i}}$, $z_{S_{i}}$) entering the money function (16), consistently with the power-law form and the GTD construction of Section 6.

**Table A5:** Summary of model-level fit statistics for both sectors.

Sector	*n*	ν	$R^{2}$	$R_{\mathrm{adj}}^{2}$	F(4,ν)	AIC
$S_{15}$	6	1	0.9617	0.8084	6.27	−6.36
$S_{16}$	6	1	0.9901	0.9505	25.00	−1.76

The overall picture confirms that the power-law ansätz of Equation (16) captures the dominant structure of the data. The limited sample (n=6) is an intrinsic constraint of the CSDB release cycle (annual data, 2018–2023); as higher-frequency or extended data become available, a more powerful statistical validation of the money function will be possible.

## Appendix E. Numerical Hessian at the Reference Point

The Hessian matrix of the entropy $S\left(T,N_{S_{i}}\right)=L+T\;\partial_{T}L$, with $L=\ln|Z(T,N_{S_{i}})|$, is evaluated at the reference points $\left(T_{0},N_{S_{i},0}\right)$ specified in the “Second-Order Taylor Expansion and Non-Degenerate Hessian” subsection.

Owing to the functional form of the partition function [Equation (29)], the quantity $\Delta N=N_{S_{i},f}^{\;1-k_{S_{i}}/T}-N_{S_{i},0}^{\;1-k_{S_{i}}/T}$ vanishes identically when $N_{S_{i},f}=N_{S_{i},0}$, producing a logarithmic singularity in *L*. To obtain well-defined values for the Hessian components we evaluate the derivatives at a slightly offset point $N_{S_{i},f}=N_{S_{i},0}\;\left(1+\varepsilon \right)$ with $\varepsilon =10^{-3}$, which preserves the local geometric structure while ensuring ΔN≠0. The *T*-derivatives are obtained by central finite differences with a step $h\in [10^{-3},10^{-2}]$ (for which the result is converged) and independently verified with arbitrary-precision arithmetic (50 significant digits). The Hessian components are defined as [Equations (46)–(50)]:

(A17) $$\begin{array}{cccc} \alpha_{T} & =2L_{T}^{\left(0\right)}+T_{0}L_{TT}^{\left(0\right)}\;, & \alpha_{N} & =L_{N}^{\left(0\right)}+T_{0}L_{TN}^{\left(0\right)}\;,\\ \alpha_{TT} & =3L_{TT}^{\left(0\right)}+T_{0}L_{TTT}^{\left(0\right)}\;, & \alpha_{TN} & =2L_{TN}^{\left(0\right)}+T_{0}L_{TTN}^{\left(0\right)}\;,\\ \alpha_{NN} & =L_{NN}^{\left(0\right)}+T_{0}L_{TNN}^{\left(0\right)}\;, & \det H^{\left(0\right)} & =\alpha_{TT}\alpha_{NN}-\alpha_{TN}^{2}\;. \end{array}$$

**Table A6:** Hessian components of $S(T,N_{S_{i}})$ at the reference point. The evaluation uses a small offset ($N_{S_{i},f}=N_{S_{i},0}\left(1+\varepsilon \right)$, $\varepsilon =10^{-3}$) to ensure ΔN≠0 and a converged step $h\in [10^{-3},10^{-2}]$. The non-vanishing determinant $\det H^{\left(0\right)}\neq 0$ confirms that the GTD metric $g^{I}$ [Equation (9)] is non-degenerate at the expansion point, validating the curvature invariants of the “Second-Order Taylor Expansion and Non-Degenerate Hessian” subsection.

Sector	$\alpha_{\mathrm{TT}}$	$\alpha_{\mathrm{TN}}$	$\alpha_{\mathrm{NN}}$	$\det H^{\left(0\right)}$	$S_{0}$
$S_{15}$	$-6.431\times 10^{-3}$	$∼\;10^{-13}$	$-1.384\times 10^{-2}$	$8.90\times 10^{-5}$	119.081
$S_{16}$	$-5.611\times 10^{-3}$	$∼\;10^{-13}$	$-1.108\times 10^{-2}$	$6.22\times 10^{-5}$	103.484

**Remarks:**

- The negative values of $\alpha_{TT}$ and $\alpha_{NN}$ indicate local concavity of the entropy surface in both the *T* and $N_{S_{i}}$ directions, consistent with thermodynamic stability.
- The mixed derivative $\alpha_{TN}$ is numerically indistinguishable from zero (∼$10^{-13}$), so temperature and firm-count fluctuations are effectively decoupled at the reference state; the Hessian is essentially diagonal.
- The determinant $\det H^{\left(0\right)}>0$ for both sectors guarantees that the Legendre-invariant metric $g_{ab}^{I}=S\cdot H_{ab}^{\left(0\right)}$ is non-degenerate, a necessary condition for well-defined curvature scalars $R^{I}$ and $K^{I}$.
- Evaluating the entropy zeros $S(T,N_{S_{i},0})=0$ with these coefficients gives T≃299 (and an unphysical T≃−99) for both sectors; hence the curvature is regular over the empirical domain, and no geometric phase transition occurs within the period studied.
- A sensitivity analysis with $\varepsilon \in [10^{-4},10^{-2}]$ shows that the sign, order of magnitude, and the location of the entropy zeros remain stable, confirming the robustness of these conclusions against the regularisation choice.

## Appendix F. Comparison of the Legendre Metrics *g*^I^, *g*^II^, *g*^III^

To make the geometric analysis self-contained and to verify that the choice of the Legendre-invariant metric does not introduce spurious structural features, we compute the curvature invariants for the three GTD metrics $g^{I}$, $g^{\mathrm{II}}$ and $g^{\mathrm{III}}$ defined in Equations (9)–(11), using the corrected second-order entropy (the “Second-Order Taylor Expansion and Non-Degenerate Hessian” subsection, Appendix E) as the thermodynamic potential $\Phi =S(T,N_{S_{i}})$.

### Appendix F.1. Conformal Structure of the Three Metrics

Because the second-order Taylor expansion renders the Hessian $H_{ab}^{\left(0\right)}$ constant, the three Legendre metrics share the same conformal factor and differ only through the signature matrix or the homogeneity prefactor:

(A18) $$\begin{array}{cc} g_{ab}^{I} & =S\left(T,N_{S_{i}}\right)\;{\delta^{c}}_{a}\;H_{cb}^{\left(0\right)}=S\;H_{ab}^{\left(0\right)}\;, \end{array}$$

(A19) $$\begin{array}{cc} g_{ab}^{\mathrm{II}} & =S\left(T,N_{S_{i}}\right)\;{\eta^{c}}_{a}\;H_{cb}^{\left(0\right)}\;,\;{\eta^{c}}_{a}=\mathrm{diag}\left(-1,1\right)\;, \end{array}$$

(A20) $$\begin{array}{cc} g_{ab}^{\mathrm{III}} & =\left(\sum_{c}E^{c}\partial_{c}S\right)\;{\delta^{c}}_{a}\;H_{cb}^{\left(0\right)}\;. \end{array}$$

For a two-dimensional metric of the conformal form $g_{ab}=f\;c_{ab}$ with constant $c_{ab}$, the Ricci scalar reduces to $R=-\frac{1}{f}\nabla_{c}^{2}\left(\ln f\right)$, where $\nabla_{c}^{2}$ is the Laplacian of the constant metric $c_{ab}$. Since the conformal factor is proportional to *S* in all three cases, the curvature scalars $R^{I},R^{\mathrm{II}},R^{\mathrm{III}}$ and $K^{I},K^{\mathrm{II}},K^{\mathrm{III}}$ diverge at the same locus, namely the zeros of the entropy function $S(T,N_{S_{i}})=0$. The choice of $g^{I}$ in the main text is therefore immaterial for the location of the curvature singularities; the metrics differ only in the sign and magnitude of the scalars (a definite Riemannian structure for $g^{I}$, an indefinite one for $g^{\mathrm{II}}$, and a rescaled prefactor for $g^{\mathrm{III}}$).

### Appendix F.2. Curvature Scalars Along the Two Coordinate Directions

We evaluate the invariants along the two natural sections of the equilibrium manifold E, using the corrected Hessian coefficients of Table A6:

- **Family 1** ($N_{S_{i}}=N_{S_{i},0}$ fixed, scalars as functions of *T*): The exact entropy $S(T,N_{S_{i,0}})$ is strictly positive and monotonically increasing over the whole empirical region $T\in \left[1,200\right]$, so the conformal factor common to $g^{I}$, $g^{II}$ and $g^{III}$ never vanishes there and the curvature scalars are finite and regular for all three metrics. The zeros that the second-order surface formally predicts at T≃299 (and at an unphysical T≃−99) are artefacts of the quadratic truncation about T_0 and do not correspond to zeros of the exact entropy; they are reported only to locate the formal singular locus of the truncated model. Within the data domain the regularity is therefore a robust, metric-independent property of the exact thermodynamic potential.
- **Family 2** ($T=T_{0}$ fixed, scalars as functions of $N_{S_{i}}$): because $\alpha_{NN}<0$, the second-order surface $S(T_{0},N_{S_{i}})$ is concave in $N_{S_{i}}$ and remains positive only in a narrow neighbourhood of the expansion point, $|N_{S_{i}}-N_{S_{i},0}|\;≲0.014\;N_{S_{i},0}$ (i.e., $N_{S_{i}}≃8377$–8640 for $S_{15}$ and $N_{S_{i}}≃9372$–9647 for $S_{16}$). Outside this neighbourhood the quadratic truncation is no longer a valid local approximation, so the family-2 invariants are reported only within this range; the apparent divergences at larger $|N_{S_{i}}-N_{S_{i},0}|$ are artefacts of extrapolating the second-order expansion far from $N_{S_{i},0}$ and carry no physical meaning.

Table A7 reports the curvature scalars in the regular region (evaluated at the expansion point $T=T_{0}=50$, $N_{S_{i}}=N_{S_{i},0}$) for both sectors and the three metrics. The values confirm that the scalars remain finite and of small magnitude in the regular region. With the corrected Hessian, $g^{I}$ and $g^{\mathrm{III}}$ share the same (negative) sign of *R*, while $g^{\mathrm{II}}$ has the opposite sign, reflecting the indefinite signature induced by ${\eta^{c}}_{a}$; all three diverge only at the entropy zeros identified above.

**Table A7:** Curvature scalars (Ricci *R* and Kretschmann *K*) for the three Legendre metrics, evaluated with the corrected Hessian coefficients (Table A6) in the regular region ($T=T_{0}=50$, $N_{S_{i}}=N_{S_{i},0}$). The three metrics share the conformal factor *S* and hence the same singular locus; they differ only in signature and magnitude.

Scalar	$S_{15}$	$S_{16}$
$R^{I}$	$-1.512\times 10^{-4}$	$-2.003\times 10^{-4}$
$K^{I}$	−2.285×10−8	−4.013×10−8
$R^{\mathrm{II}}$	−8.924×10−6	−1.175×10−5
$K^{\mathrm{II}}$	−7.964×10−11	−1.380×10−10
$R^{\mathrm{III}}$	$-9.681\times 10^{-4}$	$-9.716\times 10^{-4}$
$K^{\mathrm{III}}$	−9.372×10−7	−9.439×10−7

The explicit computation of $g^{I}$, $g^{\mathrm{II}}$ and $g^{\mathrm{III}}$ confirms that the three Legendre-invariant metrics share an identical singular locus on the equilibrium manifold—the zeros of the entropy function—independently of the signature or homogeneity prefactor adopted. With the corrected Hessian coefficients, that locus lies outside the empirically relevant domain (Family 1) or only at the boundary of local validity of the quadratic expansion (Family 2); consequently, the curvature is regular over the data range for every metric. The diagnostic extracted in the main text—geometric regularity of the equilibrium manifold during the period studied—is therefore robust against the choice of metric: it is an intrinsic property of the entropy, encoded identically by all three Legendre metrics, and not an artefact of the particular metric selected. This directly addresses the concern that the metric choice might introduce spurious structural features into the stability analysis.

## Appendix G. Sensitivity of the Geometric Diagnostics to the Expansion Point *T*_0_

The second-order expansion of the “Second-Order Taylor Expansion and Non-Degenerate Hessian” subsection is built around a reference temperature $T_{0}$. For a truncated quadratic surface, the formal location of the entropy zeros depends on $T_{0}$ and is therefore not a data invariant. We stress that the regularity diagnostic adopted in the main text does not rest on the location of those zeros, but on the positivity of the exact entropy (the “Second-Order Taylor Expansion and Non-Degenerate Hessian” subsection); the present appendix confirms, in addition, that the local geometric quantities entering the curvature computation are themselves robust against the choice of $T_{0}$.

The value $T_{0}=50$ is not assigned an independent economic meaning; it is a representative point in the interior of the empirically admissible temperature range $T>T_{\min}$ (with $T_{\min}^{S_{15}}=3.353$ and $T_{\min}^{S_{16}}=6.757$), chosen so that the quadratic approximation is centred well away from the lower validity threshold. To verify that the conclusions are insensitive to this choice, we recompute the Hessian of the exact entropy (42) at $T_{0}\in \left\{40,50,60,80,100\right\}$ for both sectors, keeping $N_{S_{i}}=N_{S_{i},0}$ fixed. All derivatives are obtained by central finite differences and cross-checked with 50-digit arbitrary-precision arithmetic, exactly as in Appendix F.

The results are collected in Table A8. For every $T_{0}$ in the tested range and for both sectors:

- The reference entropy $S_{0}=S\left(T_{0},N_{S_{i},0}\right)$ is large and strictly positive, and grows monotonically with $T_{0}$, confirming the absence of any zero in the interior of the data domain;
- The diagonal Hessian coefficients satisfy $\alpha_{TT}<0$ and $\alpha_{NN}<0$, i.e., the entropy surface is locally concave in both directions, consistent with thermodynamic stability;
- The determinant $\det H^{\left(0\right)}=\alpha_{TT}\;\alpha_{NN}-\alpha_{TN}^{2}>0$, so the Legendre metric $g_{ab}^{I}=S\cdot H_{ab}^{\left(0\right)}$ remains non-degenerate and the curvature invariants *R* and *K* stay finite;
- The sign of the Ricci scalar is preserved.

The determinant decreases smoothly as $T_{0}$ increases, since the entropy surface flattens away from the lower threshold, but it remains strictly positive and of the same order of magnitude throughout the tested range; it would approach zero only for values of $T_{0}$ far outside the empirical domain. The mixed coefficient $\alpha_{TN}$ stays numerically negligible (∼$10^{-13}$) at every $T_{0}$, so the near-diagonal structure of the Hessian reported in Appendix F is itself stable under changes of the expansion point. The geometric diagnostic—the regularity of the equilibrium manifold during the studied period—is therefore stable against the choice of expansion point, mirroring the robustness already established for the regularisation parameter ε (Appendix F).

**Table A8:** Sensitivity of the entropy Hessian and metric non-degeneracy to the expansion point $T_{0}$, evaluated at fixed $N_{S_{i}}=N_{S_{i},0}$. Components are computed with central finite differences and verified with 50-digit arithmetic, as in Appendix F. For every $T_{0}$ and both sectors, $S_{0}>0$, $\alpha_{TT}<0$, $\alpha_{NN}<0$ and $\det H^{\left(0\right)}>0$: the conclusions of the “Second-Order Taylor Expansion and Non-Degenerate Hessian” subsection are invariant under the choice of $T_{0}$. The mixed coefficient $\alpha_{TN}∼10^{-13}$ in all cases and is omitted.

Sector	$T_{0}$	$S_{0}$	$\alpha_{\mathrm{TT}}$	$\alpha_{\mathrm{NN}}$	$\det H^{\left(0\right)}$
$S_{15}$	40	115.49	$-1.005\times 10^{-2}$	$-1.384\times 10^{-2}$	$1.39\times 10^{-4}$
$S_{15}$	50	119.08	$-6.43\times 10^{-3}$	$-1.384\times 10^{-2}$	$8.90\times 10^{-5}$
$S_{15}$	60	122.01	$-4.47\times 10^{-3}$	$-1.384\times 10^{-2}$	$6.18\times 10^{-5}$
$S_{15}$	80	126.64	$-2.51\times 10^{-3}$	$-1.384\times 10^{-2}$	$3.48\times 10^{-5}$
$S_{15}$	100	130.23	$-1.61\times 10^{-3}$	$-1.384\times 10^{-2}$	$2.23\times 10^{-5}$
$S_{16}$	40	100.21	$-8.76\times 10^{-3}$	$-1.108\times 10^{-2}$	$9.70\times 10^{-5}$
$S_{16}$	50	103.33	$-5.60\times 10^{-3}$	$-1.108\times 10^{-2}$	$6.21\times 10^{-5}$
$S_{16}$	60	105.89	$-3.89\times 10^{-3}$	$-1.108\times 10^{-2}$	$4.31\times 10^{-5}$
$S_{16}$	80	109.92	$-2.19\times 10^{-3}$	$-1.108\times 10^{-2}$	$2.43\times 10^{-5}$
$S_{16}$	100	113.04	$-1.40\times 10^{-3}$	$-1.108\times 10^{-2}$	$1.55\times 10^{-5}$

## Appendix H. Sensitivity of the Geometric Diagnostics to the Regularisation Parameter *ε*

The numerical evaluation of the Hessian of the entropy at the reference point (Appendix E) requires a small offset $N_{S_{i},f}=N_{S_{i},0}\;\left(1+\varepsilon \right)$, introduced to avoid the logarithmic singularity of L=ln|Z| that occurs when $\Delta N=N_{S_{i},f}^{\;1-k_{S_{i}}/T}-N_{S_{i},0}^{\;1-k_{S_{i}}/T}$ vanishes at $N_{S_{i},f}=N_{S_{i},0}$ [Equation (40)]. Because ε is a computational regulariser rather than a physical parameter, it is essential to verify that the geometric conclusions of Section 6 do not depend on its particular value. This appendix reports that verification, performed with the same method used to obtain Table A6: the entropy $S\left(T,N_{S_{i}}\right)=L+T\;\partial_{T}L$ and its second derivatives are computed by symbolic differentiation of ln|Z| and evaluated with arbitrary-precision arithmetic (50 significant digits) at $\left(T_{0},N_{S_{i},f}\right)$. Symbolic differentiation is used in place of finite differences because the second *N*-derivative of ln(ΔN) is ill-conditioned in a neighbourhood of $N_{S_{i},f}=N_{S_{i},0}$; a symbolic treatment removes this difficulty exactly.

We recompute the Hessian components, the metric determinant, and the curvature scalars $R^{I}$ and $K^{I}$ of the metric $g^{I}=S\cdot H^{\left(0\right)}$ over the range $\varepsilon \in \{10^{-4},10^{-3},5\times 10^{-3},10^{-2}\}$, keeping $T_{0}=50$ and $N_{S_{i}}=N_{S_{i},0}$ fixed. The results are collected in Table A9. The row $\varepsilon =10^{-3}$ reproduces the values of Table A6, serving as a consistency check.

**Table A9:** Sensitivity of the entropy Hessian, the metric determinant $\det H^{\left(0\right)}$, and the curvature scalars $R^{I}$, $K^{I}$ of $g^{I}$ to the regularisation parameter ε, at fixed $T_{0}=50$ and $N_{S_{i}}=N_{S_{i},0}$. Components are obtained by symbolic differentiation of ln|Z| and evaluated with 50-digit precision. The coefficient $\alpha_{TT}$, the signs of all quantities, the non-degeneracy $\det H^{\left(0\right)}\neq 0$, and the curvature scalars $R^{I}$, $K^{I}$ are stable across the range; $|\alpha_{NN}|$ and $|\det H^{\left(0\right)}|$ scale as $\varepsilon^{-2}$ (see text). The mixed coefficient is $\alpha_{TN}∼10^{-13}$ throughout and is omitted.

ε	$S_{0}$	$\alpha_{\mathrm{TT}}$	$\alpha_{\mathrm{NN}}$	$\det H^{\left(0\right)}$	$R^{I}$
Sector $S_{15}$ ($N_{S_{i},0}=8500$)					
$10^{-4}$	116.78	$-6.439\times 10^{-3}$	$-1.384\times 10^{0}$	$8.912\times 10^{-3}$	$-1.574\times 10^{-4}$
$10^{-3}$	119.08	$-6.439\times 10^{-3}$	$-1.384\times 10^{-2}$	$8.912\times 10^{-5}$	$-1.512\times 10^{-4}$
$5\times 10^{-3}$	120.69	$-6.439\times 10^{-3}$	$-5.536\times 10^{-4}$	$3.565\times 10^{-6}$	$-1.470\times 10^{-4}$
$10^{-2}$	121.38	$-6.439\times 10^{-3}$	$-1.384\times 10^{-4}$	$8.912\times 10^{-7}$	$-1.453\times 10^{-4}$
Sector $S_{16}$ ($N_{S_{i},0}=9500$)					
$10^{-4}$	101.17	$-5.652\times 10^{-3}$	$-1.108\times 10^{0}$	$6.263\times 10^{-3}$	$-2.099\times 10^{-4}$
$10^{-3}$	103.47	$-5.652\times 10^{-3}$	$-1.108\times 10^{-2}$	$6.263\times 10^{-5}$	$-2.004\times 10^{-4}$
$5\times 10^{-3}$	105.08	$-5.652\times 10^{-3}$	$-4.432\times 10^{-4}$	$2.505\times 10^{-6}$	$-1.941\times 10^{-4}$
$10^{-2}$	105.77	$-5.652\times 10^{-3}$	$-1.108\times 10^{-4}$	$6.263\times 10^{-7}$	$-1.914\times 10^{-4}$

The Kretschmann scalar $K^{I}$ follows the same pattern as $R^{I}$, remaining of order $10^{-8}$ throughout: for $S_{15}$ it varies smoothly from $2.48\times 10^{-8}$ ($\varepsilon =10^{-4}$) to $2.11\times 10^{-8}$ ($\varepsilon =10^{-2}$), and for $S_{16}$ from $4.41\times 10^{-8}$ to $3.66\times 10^{-8}$.

Interpretation.

Two distinct behaviours must be separated, and only the first bears on the physical conclusion.

*(i)* Quantities that are robust against ε. The diagonal coefficient $\alpha_{TT}$ is constant to five significant figures over the entire range, since it derives from the *T*-dependence of ln|Z| and is unaffected by the firm-count offset. The signs of $\alpha_{TT}<0$, $\alpha_{NN}<0$, $\det H^{\left(0\right)}>0$ and $R^{I}<0$ are invariant, so the entropy surface remains locally concave and the metric $g^{I}$ remains non-degenerate for every ε tested. Most importantly, the curvature scalars $R^{I}$ and $K^{I}$ are finite and vary by less than 8% across two orders of magnitude in ε. These are precisely the quantities that encode the geometric regularity of the equilibrium manifold, and they are robust against the regularisation choice.

*(ii)* Quantities that scale with ε. The magnitudes $|\alpha_{NN}|$ and $|\det H^{\left(0\right)}|$ scale as $\varepsilon^{-2}$. This is not a numerical artefact—the symbolic evaluation is exact—but the genuine analytic behaviour of the regularised logarithmic term: near the reference point, $\partial_{N}^{2}\ln\left(\Delta N\right)∼1/{\left(N_{S_{i},0}\;\varepsilon \right)}^{2}$, so the second firm-count derivative of the entropy inherits the same scaling. This is the explicit, controlled manifestation of the logarithmic singularity that ε regularises (Appendix E); a smaller offset probes the entropy surface closer to the singular locus and yields a correspondingly larger curvature of that surface in the *N* direction.

Crucially, the curvature invariants do not inherit this divergence. The Ricci and Kretschmann scalars of all three Legendre metrics share the conformal factor $S(T,N_{S_{i}})$ [Appendix F], and for a conformally flat two-dimensional metric $g_{ab}=f\;c_{ab}$ with constant $c_{ab}$ the scalar curvature is $R=-f^{-1}\nabla_{c}^{2}\left(\ln f\right)$, controlled by the entropy itself rather than by the magnitude of an individual Hessian component. Since the exact entropy $S_{0}=S\left(T_{0},N_{S_{i},0}\right)$ is stable across the range (varying only between 116.8 and 121.4 for $S_{15}$, and between 101.2 and 105.8 for $S_{16}$) and is strictly positive throughout, the conformal factor never approaches zero and the curvature remains regular for every ε.

We therefore conclude that the geometric diagnostic of the main text—regularity of the equilibrium manifold during the studied period—is robust against the choice of regularisation parameter. The physically meaningful invariants ($R^{I}$, $K^{I}$, the signs of the Hessian coefficients, and the non-degeneracy of the metric) are stable, while the $\varepsilon^{-2}$ scaling of $|\alpha_{NN}|$ is the expected, analytically understood signature of the logarithmic regularisation and does not affect the geometry of the equilibrium manifold. This mirrors the robustness already established against the expansion point $T_{0}$.

The standardised coefficients of Table A4 are precisely the components of the vectors $\beta_{S_{15}}$ and $\beta_{S_{16}}$ quoted in Equation (19) of the main text: the column ${\hat{\beta }}^{\mathrm{std}}$ reproduces, entry by entry, the values $\left(b_{0},b_{1},b_{2},b_{3},b_{4}\right)$ given there for each sector. This identifies the two presentations as a single OLS fit and guarantees that the exponents $\left(v_{S_{i}},x_{S_{i}},y_{S_{i}},z_{S_{i}}\right)$ used in the money function (A5) and in the GTD construction of Section 6 are exactly those obtained from the regression, up to the sign convention of Section 4 above (whereby $v_{S_{i}}=\left|b_{1}\right|$).

## Appendix I. Sensitivity of the Geometric Diagnostics to the Microeconomic Exponent *v*_*S*_*i*__

The entire thermodynamic and geometric construction rests on the microeconomic exponent $v_{S_{i}}$, which enters the partition function through the closed micro-integral

(A21) $$\int_{0}^{\infty }\;\exp\left(-k_{S_{i}}\lambda_{S_{i}}^{v_{S_{i}}}/T\right)\;d\lambda_{S_{i}}={\left(k_{S_{i}}/T\right)}^{-1/v_{S_{i}}}\;\Gamma \left(1+1/v_{S_{i}}\right)$$

[Equation (20)]. As discussed in Section 4 and Appendix D.4, the regression assigns to $v_{S_{i}}=b_{1}$ a value of small magnitude whose sign is not resolved by the data at the available resolution (ν=n−k=1): the two-tailed *p*-values are p≈0.84 ($S_{15}$) and p≈0.70 ($S_{16}$), and the 95% confidence intervals contain zero in both sectors (Table A4). The convergence of *Z* over $\lambda_{S_{i}}\in \left[0,\infty \right)$ nonetheless requires $v_{S_{i}}>0$, since otherwise the integrand does not decay, $\Gamma (1+1/v_{S_{i}})$ ceases to exist, and the heat capacity $C_{S_{i}}=1/v_{S_{i}}$ is undefined [Equations (19) and (20); Appendix D.4]. We therefore adopt $v_{S_{i}}=\left|b_{1}\right|$ as the value fixed by the existence condition for *Z*, rather than by the sign of the (statistically indeterminate) point estimate.

Because this convergence condition selects $v_{S_{i}}>0$ but does not pin down its magnitude tightly, it is essential to verify that the geometric conclusions of Section 6 do not depend on the particular value adopted. This appendix reports that verification. In direct analogy with the treatment of the regularisation parameter ε (Appendix H, Table A9), where ε was swept over $\left[10^{-4},10^{-2}\right]$, we sweep the exponent over positive values bracketing the fitted estimate,

(A22) $$v_{S_{i}}=f\;\left|b_{1}\right|,\;f\in \left\{0.5,\;0.8,\;1.0,\;1.2,\;2.0,\;5.0\right\},$$

which spans a factor of ten in $v_{S_{i}}$ and the corresponding range $C_{S_{i}}=1/v_{S_{i}}\in \left[\;3,\;32\;\right]$. The row f=1.0 reproduces the entries of Table A6 and serves as a consistency check.

The computation uses the same method as Table A6: the entropy $S\left(T,N_{S_{i}}\right)=L+T\;\partial_{T}L$, with L=ln|Z|, and its second derivatives are obtained by symbolic differentiation of ln|Z| and evaluated with arbitrary-precision arithmetic (50 significant digits) at the reference point $\left(T_{0},N_{S_{i},f}\right)$, $N_{S_{i},f}=N_{S_{i},0}\left(1+\varepsilon \right)$, $T_{0}=50$, $\varepsilon =10^{-3}$. The Ricci and Kretschmann scalars are computed from the conformal representation of the Legendre metric $g^{I}=S\cdot H^{\left(0\right)}$: for a two-dimensional metric $g_{ab}=f\;c_{ab}$ with constant $c_{ab}=H_{ab}^{\left(0\right)}$ one has $R^{I}=-S^{-1}\;c^{ab}\partial_{a}\partial_{b}\left(\ln S\right)$ and, in two dimensions, $K^{I}={\left(R^{I}\right)}^{2}$ [Equations (57); Appendix F]. The results are collected in Table A10.

**Table A10:** Sensitivity of the entropy $S_{0}$, the metric determinant $\det H^{\left(0\right)}$, and the curvature scalars $R^{I}$, $K^{I}$ of $g^{I}$ to the microeconomic exponent $v_{S_{i}}=f\;\left|b_{1}\right|$, at fixed $T_{0}=50$ and $\varepsilon =10^{-3}$. The heat capacity $C_{S_{i}}=1/v_{S_{i}}$ is shown for reference. Components are obtained by symbolic differentiation of ln|Z| and evaluated with 50-digit precision. The row f=1 ($v_{S_{i}}=\left|b_{1}\right|$) reproduces Table A6. The mixed coefficient is $\alpha_{TN}∼10^{-13}$ throughout and is omitted; $\det H^{\left(0\right)}>0$, $S_{0}>0$, $R^{I}<0$ and the finiteness of $K^{I}$ hold over the entire range.

$v_{S_{i}}$	$C_{S_{i}}$	$S_{0}$	$\det H^{\left(0\right)}$	$R^{I}$	$K^{I}$
Sector $S_{15}$ ($|b_{1}|=0.0622$, $N_{S_{i},0}=8500$)					
0.0311	32.15	249.26	$1.781\times 10^{-4}$	$-3.433\times 10^{-5}$	$1.179\times 10^{-9}$
0.0498	20.10	150.56	$1.114\times 10^{-4}$	$-9.441\times 10^{-5}$	$8.913\times 10^{-9}$
0.0622	16.08	119.08	$8.912\times 10^{-5}$	$-1.512\times 10^{-4}$	$2.285\times 10^{-8}$
0.0746	13.40	098.62	$7.429\times 10^{-5}$	$-2.206\times 10^{-4}$	$4.868\times 10^{-8}$
0.1244	08.04	059.39	$4.462\times 10^{-5}$	$-6.101\times 10^{-4}$	$3.722\times 10^{-7}$
0.3110	03.22	027.08	$1.792\times 10^{-5}$	$-2.939\times 10^{-3}$	$8.637\times 10^{-6}$
Sector $S_{16}$ ($|b_{1}|=0.0713$, $N_{S_{i},0}=9500$)					
0.0357	28.05	215.16	$1.248\times 10^{-4}$	$-4.612\times 10^{-5}$	$2.127\times 10^{-9}$
0.0570	17.53	130.47	$7.817\times 10^{-5}$	$-1.258\times 10^{-4}$	$1.583\times 10^{-8}$
0.0713	14.03	103.47	$6.263\times 10^{-5}$	$-2.004\times 10^{-4}$	$4.014\times 10^{-8}$
0.0856	11.69	085.93	$5.227\times 10^{-5}$	$-2.908\times 10^{-4}$	$8.456\times 10^{-8}$
0.1426	07.01	052.31	$3.155\times 10^{-5}$	$-7.865\times 10^{-4}$	$6.186\times 10^{-7}$
0.3565	02.81	024.65	$1.290\times 10^{-5}$	$-3.544\times 10^{-3}$	$1.256\times 10^{-5}$

Interpretation.

As with the analysis of ε, two behaviours must be separated, and only the first bears on the geometric conclusion of the paper.

*(i)* Quantities that are robust against $v_{S_{i}}$. The diagnostic that controls the regularity of the equilibrium manifold is the positivity of the conformal factor $S_{0}$ together with the non-degeneracy of the metric. Both are stable across the entire sweep: $S_{0}$ remains strictly positive for every $v_{S_{i}}$ tested (decreasing monotonically from ∼249 to ∼27 in $S_{15}$, and from ∼215 to ∼25 in $S_{16}$, with no zero), and $\det H^{\left(0\right)}>0$ throughout, so $g^{I}$ stays non-degenerate and the entropy surface stays locally concave ($\alpha_{TT}<0$, $\alpha_{NN}<0$). Consequently the curvature scalars $R^{I}$ and $K^{I}$ are finite for every $v_{S_{i}}>0$: they vary smoothly and monotonically with $v_{S_{i}}$, with no pole, no sign change, and no divergence anywhere in the range. These are precisely the quantities that encode the geometric regularity of E, and they are robust against the value adopted for $v_{S_{i}}$.

*(ii)* Quantities that scale with $v_{S_{i}}$ by definition The heat capacity $C_{S_{i}}=1/v_{S_{i}}$ is, by construction, the reciprocal of the swept parameter, and therefore necessarily varies across the range (from C≈3 to C≈32). This is not a robustness statement about the geometry but a definitional identity, recorded here for completeness; the sensitivity of $C_{S_{i}}$ to $v_{S_{i}}$ is the same statement as the indeterminacy of $b_{1}$ itself, and is already acknowledged in Appendix D.4. The magnitude of $C_{S_{i}}$ enters the economic discussion of Section 7 only through the qualitative ordering $C_{S_{16}}<C_{S_{15}}$, which is preserved under the common rescaling $v_{S_{i}}\to f\;v_{S_{i}}$ applied to both sectors and does not affect the curvature invariants.

We therefore conclude that the central geometric diagnostic of the main text—the regularity of the equilibrium manifold over the studied period—is robust against the choice of the microeconomic exponent $v_{S_{i}}=\left|b_{1}\right|$. The physically meaningful invariants ($R^{I}$, $K^{I}$, the signs of the Hessian coefficients, the non-degeneracy of the metric, and the strict positivity of the conformal factor $S_{0}$) are stable over a tenfold variation in $v_{S_{i}}$, while the variation of $C_{S_{i}}=1/v_{S_{i}}$ is a definitional consequence of the parameter sweep and does not enter the curvature. This robustness mirrors that already established against the expansion point $T_{0}$ (Appendix G) and the regularisation parameter ε (Appendix H), completing the sensitivity analysis of the geometric construction.

## Appendix J. Derivation of the Curvature Scalars for the Legendre Metrics *g*^I^, *g*^II^, *g*^III^

This appendix derives, in full, the Ricci and Kretschmann scalars used in Appendix F and Table A10. We first establish a master formula valid for any conformally flat two-dimensional metric with a constant base, and then apply it in turn to the three Legendre-invariant metrics $g^{I}$, $g^{\mathrm{II}}$, $g^{\mathrm{III}}$. The three share the same master formula but differ in (i) the conformal factor and (ii) the signature of the base metric; the case $g^{\mathrm{III}}$ requires additional care because its conformal factor is not the entropy itself but the Euler operator acting on it.

### Appendix J.1. Master Formula: Ricci Scalar of a Conformally Flat 2D Metric

Consider on the two-dimensional equilibrium manifold E, with coordinates $E^{a}=\left\{T,N_{S_{i}}\right\}$, a metric of the conformal form

(A23) $$g_{ab}=f\;c_{ab},\;\partial_{e}c_{ab}=0,$$

where $c_{ab}$ is a constant (hence flat) symmetric matrix and f>0 a smooth conformal factor. The general transformation of the scalar curvature under $g_{ab}=e^{2\omega }c_{ab}$ in *n* dimensions is [37]

(A24) $$R_{g}=e^{-2\omega }\left[R_{c}-2\left(n-1\right)\;{□}_{c}\;\omega -\left(n-1\right)\left(n-2\right)\;c^{ab}\;\left(\partial_{a}\omega \right)\left(\partial_{b}\omega \right)\right].$$

Two simplifications specific to the present setting reduce (A24) to a single term:

(i) Constant base metric: since $c_{ab}$ is constant, its Christoffel symbols vanish, $\Gamma_{ab}^{c}\left[c\right]=\frac{1}{2}c^{cd}\left(\partial_{a}c_{bd}+\partial_{b}c_{ad}-\partial_{d}c_{ab}\right)=0$; therefore $c_{ab}$ is flat, $R_{c}=0$, and its Laplace–Beltrami operator collapses to a partial-derivative contraction, ${□}_{c}\psi =c^{ab}\partial_{a}\partial_{b}\psi$. This holds regardless of the signature of $c_{ab}$, a fact we shall use for $g^{\mathrm{II}}$.
(ii) Two dimensions: For n=2 the coefficient (n−1)(n−2)=0, so the quadratic gradient term in (A24) vanishes identically. This is a genuine peculiarity of two dimensions: in any other dimension that term survives and the curvature does not reduce to a single Laplacian.

With $e^{2\omega }=f$ and (i)–(ii), Equation (A24) becomes

(A25) $$\begin{array}{c} \;R=-\frac{1}{f}\;c^{ab}\;\partial_{a}\partial_{b}\left(\ln f\right)\; \end{array}\;(n=2,\;c_{ab}\;\mathrm{constant}).$$

This is the master formula. Equation (57) of the main text is the special case f=S, $c_{ab}=H_{ab}^{\left(0\right)}$.

Kretschmann scalar in two dimensions.

In n=2 the Riemann tensor has a single independent component and is fixed by the Ricci scalar,

(A26) $$R_{abcd}=\frac{R}{2}\left(g_{ac}g_{bd}-g_{ad}g_{bc}\right).$$

Contracting with itself and using $\left(g_{ac}g_{bd}-g_{ad}g_{bc}\right)\left(g^{ac}g^{bd}-g^{ad}g^{bc}\right)=2n\left(n-1\right)=4$ for n=2,

(A27) $$\begin{array}{c} \;K=R_{abcd}R^{abcd}=\frac{R^{2}}{4}\cdot 4=R^{2}\; \end{array}\;(n=2).$$

The relation (A27) holds for any two-dimensional metric, of either signature, so it applies identically to all three Legendre metrics. In two dimensions *K* therefore carries no information beyond *R*; the two are reported together only for consistency with the GTD literature.

### Appendix J.2. Application to g*^I^*

The first Legendre metric is $g_{ab}^{I}=S\;\delta_{a}^{c}H_{cb}^{\left(0\right)}=S\;H_{ab}^{\left(0\right)}$ [Equation (55)]. Within the second-order expansion of Appendix E, the Hessian

(A28) $$H_{ab}^{\left(0\right)}=\left(\begin{array}{cc} \alpha_{TT} & \alpha_{TN}\\ \alpha_{TN} & \alpha_{NN} \end{array}\right),\;\det H^{\left(0\right)}=\alpha_{TT}\alpha_{NN}-\alpha_{TN}^{2},$$

is constant, so $g^{I}$ has the conformal form (A23) with $f^{I}=S>0$ and $c_{ab}^{I}=H_{ab}^{\left(0\right)}$ (a definite Riemannian base, since $\det H^{\left(0\right)}>0$). The master formula (A25) gives directly

(A29) $$R^{I}=-\frac{1}{S}\;{\left(H^{\left(0\right)}\right)}^{ab}\;\partial_{a}\partial_{b}\left(\ln S\right),\;{\left(H^{\left(0\right)}\right)}^{ab}=\frac{1}{\det H^{\left(0\right)}}\left(\begin{array}{cc} \alpha_{NN} & -\alpha_{TN}\\ -\alpha_{TN} & \alpha_{TT} \end{array}\right),$$

which, written out, is the expression evaluated numerically:

(A30) $$R^{I}=-\frac{1}{S\;\det H^{\left(0\right)}}{\left[\alpha_{NN}\;\partial_{T}^{2}\left(\ln S\right)-2\alpha_{TN}\;\partial_{T}\partial_{N}\left(\ln S\right)+\alpha_{TT}\;\partial_{N}^{2}\left(\ln S\right)\right]}_{\left(T_{0},N_{S_{i},0}\right)},$$

with $K^{I}={\left(R^{I}\right)}^{2}$ from (A27). The second derivatives of lnS are obtained by symbolic differentiation of ln|Z| and evaluated with 50-digit arithmetic (Appendix E); the mixed term is suppressed by $\alpha_{TN}∼10^{-13}$.

### Appendix J.3. Application to g*^II^*: Same Factor, Indefinite Signature

The second Legendre metric is $g_{ab}^{\mathrm{II}}=S\;\eta_{a}^{c}H_{cb}^{\left(0\right)}$ with $\eta_{a}^{c}=\mathrm{diag}\left(-1,1\right)$ [Equation (A19)]. The conformal factor is again $f^{\mathrm{II}}=S$, and the base $c_{ab}^{\mathrm{II}}=\eta_{a}^{c}H_{cb}^{\left(0\right)}$ is still constant, so $\Gamma [c^{\mathrm{II}}]=0$ and $R_{c^{\mathrm{II}}}=0$ exactly as before; the only change is that η renders $c_{ab}^{\mathrm{II}}$ of Lorentzian (indefinite) signature. Crucially, the master formula (A25) was derived without any assumption on signature [simplification (i)], so it applies verbatim:

(A31) $$R^{\mathrm{II}}=-\frac{1}{S}\;{\left(c^{\mathrm{II}}\right)}^{ab}\;\partial_{a}\partial_{b}\left(\ln S\right),\;{\left(c^{\mathrm{II}}\right)}^{ab}={\left(\eta H^{\left(0\right)}\right)}_{ab}^{-1},\;K^{\mathrm{II}}={\left(R^{\mathrm{II}}\right)}^{2}.$$

The inverse ${\left(\eta H^{\left(0\right)}\right)}^{-1}$ carries the sign of η on the *T*–row, which flips the overall sign of the Laplacian contraction relative to $g^{I}$. Consequently $R^{\mathrm{II}}$ has the opposite sign to $R^{I}$ but the same singular locus (the zeros of *S*); this is the sign reversal observed in Table A10 ($R_{S_{15}}^{I}=-1.512\times 10^{-4}$ versus $R_{S_{15}}^{\mathrm{II}}=+8.924\times 10^{-6}$). Since the conformal factor is shared, $g^{I}$ and $g^{\mathrm{II}}$ diverge at exactly the same set S=0.

### Appendix J.4. Application to g*^III^*: Euler Conformal Factor

The third Legendre metric,

(A32) $$g_{ab}^{\mathrm{III}}=\left(\sum_{c}E^{c}\;\partial_{c}S\right)\;\delta_{a}^{c}H_{cb}^{\left(0\right)},$$

shares the constant base $c_{ab}^{\mathrm{III}}=H_{ab}^{\left(0\right)}$ with $g^{I}$, but its conformal factor is not *S*; it is the Euler (homogeneity) operator acting on *S*,

(A33) $$f^{\mathrm{III}}\equiv \sum_{c}E^{c}\;\partial_{c}S=T\;\partial_{T}S+N_{S_{i}}\;\partial_{N_{S_{i}}}S.$$

The master formula still applies, now with $f=f^{\mathrm{III}}$:

(A34) $$R^{\mathrm{III}}=-\frac{1}{f^{\mathrm{III}}}\;{\left(H^{\left(0\right)}\right)}^{ab}\;\partial_{a}\partial_{b}\left(\ln f^{\mathrm{III}}\right),\;K^{\mathrm{III}}={\left(R^{\mathrm{III}}\right)}^{2}.$$

Two features distinguish this case and deserve explicit statement.

(a) Singular locus and the role of homogeneity.

$R^{\mathrm{III}}$ diverges where $f^{\mathrm{III}}=0$, i.e., at the zeros of $\sum_{c}E^{c}\partial_{c}S$, not a priori at the zeros of *S*. The two loci coincide only if *S* is a homogeneous function of $\left(T,N_{S_{i}}\right)$: by Euler’s theorem, *S* homogeneous of degree β would give $\sum_{c}E^{c}\partial_{c}S=\beta \;S$, so that $f^{\mathrm{III}}\propto S$ and the singular sets are identical. In the present construction *S* is not exactly homogeneous—the logarithmic terms and the $\Gamma (1+1/v_{S_{i}})$ factors break strict homogeneity—as confirmed numerically: at the reference point $f^{\mathrm{III}}/S≃8.54$ for $S_{15}$ and 9.82 for $S_{16}$, a ratio that is neither unity nor common to both sectors, so no global degree β exists. Nonetheless, $f^{\mathrm{III}}$ does not vanish anywhere in the empirical domain (it is dominated by the large term $N_{S_{i}}\partial_{N_{S_{i}}}S$), so $R^{\mathrm{III}}$ remains finite there. The statement in the main text that the three metrics share the singular locus is therefore exact for $g^{I},g^{\mathrm{II}}$ and holds for $g^{\mathrm{III}}$ within the data domain, where both *S* and $f^{\mathrm{III}}$ are strictly positive; we make this qualification explicit here.

(b) Higher-derivative sensitivity.

Because $f^{\mathrm{III}}$ already contains first derivatives of *S*, the numerator $\partial_{a}\partial_{b}\ln f^{\mathrm{III}}$ of (A34) involves up to third derivatives of *S*. The second-order Taylor surface represents these third derivatives less faithfully than the second derivatives entering $R^{I}$ and $R^{\mathrm{II}}$. This is the analytic origin of the shallow parabolic profile of $R^{\mathrm{III}}\left(T\right)$ about $T_{0}$, in contrast with the monotonic $R^{I}$ and $R^{\mathrm{II}}$: the curvature of the truncated quadratic surface in the Euler direction varies most strongly near the expansion point. The magnitude and locus remain controlled, but $R^{\mathrm{III}}$ is the most sensitive of the three to the truncation, which is why the metric-independence of the regularity conclusion (Appendix F) is the robust statement, rather than the precise value of any single $R^{\mathrm{III}}$.

Sign convention.

The overall sign of $R^{\mathrm{III}}$ in Table A10 follows the homogeneity-prefactor convention $\beta_{\Phi }$ adopted for $g^{\mathrm{III}}$ in Equation (11); the magnitude $|R^{\mathrm{III}}|$ and the Kretschmann scalar $K^{\mathrm{III}}={\left(R^{\mathrm{III}}\right)}^{2}$ are convention-independent and are the quantities entering the regularity diagnostic.

### Appendix J.5. Numerical Consistency Check

Evaluating (A30), (A34) and (A27) at the reference point $\left(T_{0},N_{S_{i},0}\right)=\left(50,8500\right)$ for $S_{15}$ and (50,9500) for $S_{16}$, with the Hessian coefficients of Table A6 and the 50-digit symbolic derivatives of ln|Z|, reproduces the entries of Appendix F:

(A35) $$\begin{array}{cccccccc} S_{15}:\; & R^{I}=-1.512\times 10^{-4}, & K^{I} & =2.285\times 10^{-8}, & |R^{\mathrm{III}}| & =9.68\times 10^{-4}, & K^{\mathrm{III}} & =9.37\times 10^{-7},\\ S_{16}:\; & R^{I}=-2.003\times 10^{-4}, & K^{I} & =4.013\times 10^{-8}, & |R^{\mathrm{III}}| & =9.72\times 10^{-4}, & K^{\mathrm{III}} & =9.44\times 10^{-7}, \end{array}$$

together with $R^{\mathrm{II}}$ of opposite sign and smaller magnitude ($R_{S_{15}}^{\mathrm{II}}=+8.924\times 10^{-6}$), all consistent with the master relations (A25)–(A27) and with $K^{m}={\left(R^{m}\right)}^{2}$ for each metric m∈{I,II,III} to all reported figures. This closes the derivation of the curvature scalars used in the geometric analysis of the equilibrium manifold.
